# Increased Blood Flow and Tendon Swelling Precedes Vascular Expansion and Tissue Matrix Changes In Early Human Tendinopathy: A Potential Window for Superior Treatment Response

**DOI:** 10.1002/advs.202514023

**Published:** 2025-12-12

**Authors:** Max F. R. Merkel, Nikolaj M. Malmgaard‐Clausen, Marius Lendal, Hartwig R. Siebner, René B. Svensson, Stephanie G. Dakin, Marcus Krüger, Luisa Schmidt, Jakob Agergaard, Ching‐Yan Chloé Yeung, S. Peter Magnusson, Michael Kjaer

**Affiliations:** ^1^ Institute of Sports Medicine Copenhagen Department of Orthopedic Surgery Copenhagen University Hospital – Bispebjerg and Frederiksberg Copenhagen 2400 Denmark; ^2^ Department of Clinical Medicine University of Copenhagen Copenhagen 2200 Denmark; ^3^ Danish Research Centre for Magnetic Resonance Department of Radiology and Nuclear Medicine Copenhagen University Hospital‐Amager and Hvidovre Copenhagen 2650 Denmark; ^4^ Department of Neurology Copenhagen University Hospital Bispebjerg and Frederiksberg Copenhagen 2400 Denmark; ^5^ Botnar Institute for Musculoskeletal Sciences Nuffield Department of Orthopaedics, Rheumatology and Musculoskeletal Sciences University of Oxford Oxford OX3 7LD UK; ^6^ NIHR Oxford Biomedical Research Unit Botnar Research Centre University of Oxford Oxford OX3 7LD UK; ^7^ Institute for Genetics Cologne Excellence Cluster on Cellular Stress Responses in Aging‐Associated Diseases (CECAD) University of Cologne 50931 Cologne Germany; ^8^ Center for Molecular Medicine (CMMC) University of Cologne 50931 Cologne Germany; ^9^ Novo Nordisk Foundation Center for Protein Research Department of Cellular and Molecular Medicine Faculty of Health and Medical Sciences University of Copenhagen Copenhagen 2200 Denmark; ^10^ Center for Fast Ultrasound Imaging Department of Health Technology Technical University of Denmark Lyngby 2800 Denmark

**Keywords:** angiogenesis, blood flow, early tendinopathy, tendinopathy, tendon swelling

## Abstract

Tendinopathy represents a major musculoskeletal health problem, yet its pathogenesis remains poorly understood. Tendinopathy development is studied in humans with early (< 3 months of symptoms, *n* = 14) (ET) or chronic (> 3 months, *n* = 16) (CT) patellar tendinopathy and in healthy subjects (*n* = 15) (CTRL). Pain increases, and function declines with tendinopathy duration and correlated with tendon size (3T and 7T MRI). Tendon blood flow (Doppler ultrasonography) increases gradually in ET and CT, while peritendinous blood flow only rose in CT. Microscopy‐based mapping (immunofluorescence microscopy and Cell DIVE) of vasculature shows marked changes in CT only, indicating flow increases in existing vessels early in tendinopathy, whereas angiogenesis is a late phenomenon. Cell DIVE indicates perivascular cell recruitment and potential lymphatic expansion in tendinopathy. Further, proteomics reveals that most matrix regulation occurs late in tendinopathy. Data from a previous study from the lab demonstrate faster treatment effect in tendinopathy with shorter symptom duration, supporting that early tissue changes may be more receptive to treatment. It is concluded that early tendinopathy is dominated by pain correlating with tendon swelling and hyperperfusion, whereas chronic tendinopathy is characterized by neovascularization and matrisome changes. These findings suggest that targeting early tissue changes can lead to superior treatment effects in tendinopathy.

## Graphical abstract

Human participants with early‐ or chronic‐stage tendinopathy and healthy controls are investigated using ultrasound Doppler, 3T and 7T MRI, and tendon biopsies. Tendon swelling and hyperperfusion are evident early, while vascular growth and extracellular matrix changes are most pronounced in chronic tendinopathy. Early intervention may provide superior treatment outcomes before persistent tissue changes manifest in chronic tendinopathy.

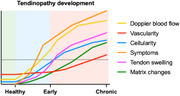

## 1 Introduction

Tendinopathy is a tendon disorder characterized by pain during physical activity, focal tenderness upon palpation, and impaired performance in both sports and daily life^[^ ^1^, ^2^ ^]^ Despite the fact that the socio‐economic burden of tendinopathy is substantial,^[^ ^3^ ^]^ the pathogenesis and development of the condition remain poorly understood.^[^ ^4^ ^]^ Clinical symptoms in tendinopathy are associated with tendon swelling, increased tendon blood flow, as well as structural and cellular changes in the matrix affecting collagen organization and composition.^[^ ^4^, ^5^ ^]^ However, the sequence of these events is unknown.

Non‐invasive imaging modalities like magnetic resonance imaging (MRI) and ultrasonography (US) have been used to describe thickening of the tendon in tendinopathy. US can be used to estimate tendon blood blow using Doppler, and MRI is indirectly able to detail both structural changes and estimate increased water content in the tendinopathic tendon.^[^ ^6^, ^7^, ^8^ ^]^ Despite these advances in tendon imaging, we still know little about how structural and vascular changes are related, and it is currently unknown how early water accumulation and swelling occur with tendinopathy. Several studies have investigated the relationship between tendon neovascularization and clinical symptoms of tendinopathy,^[^ ^9^, ^10^, ^11^, ^12^ ^]^ but no clear relationship has been established.^[^ ^13^ ^]^ Newer studies indicate that tendon pain might relate to the accumulation of proteoglycans (PG) and glycosaminoglycans (GAG) in the tendon tissue,^[^ ^14^, ^15^ ^]^ and that associated water accumulation leads to elevated intratendinous pressure.^[^ ^16^ ^]^ However, the particular time patterns for these factors remain to be described. Further, since US Doppler is not able to identify individual blood vessels, it remains undetermined if the increased Doppler flow represents vascular growth, altered perfusion dynamics in existing vessels, or both.

Microscopy‐based studies of tendinopathy have addressed the role of inflammation,^[^ ^17^, ^18^, ^19^ ^]^ cellular proliferation and matrix disorganization,^[^ ^20^ ^]^ as well as neurovascular ingrowth and nociceptive tissue substances.^[^ ^21^ ^]^ However, evidence remains conflicting as to whether inflammation is increased in tendinopathy.^[^ ^22^, ^23^, ^24^ ^]^ While cell numbers and phenotypes are altered,^[^ ^25^, ^26^ ^]^ the underlying cellular processes and consequent protein changes in the tendon tissue remain unclear.

The purpose of the current study was to characterize tendinopathy pathogenesis and development in humans. Specifically, we compared human patellar tendons from individuals with early tendinopathy (1–2 months duration), chronic tendinopathy (3 months to 10 years duration), and healthy individuals. Clinical symptoms were assessed using questionnaires and physical examinations, tendon size was evaluated using 3‐tesla (3T) and 7T MRI as well as US, and tendon blood flow was measured via US Doppler. To understand changes in tendon matrix and spatial morphology in depth, microscopy‐based (traditional immunofluorescence and Cell DIVE) and proteomic analyses were conducted on human tendon biopsies. We hypothesized that tendon swelling and increased blood perfusion are early events in tendinopathy, while in contrast, changes in tissue protein and morphology occur in the later stage of tendinopathy.

## 2 Results

### 2.1 Subject Characteristics

**Figure 1:**
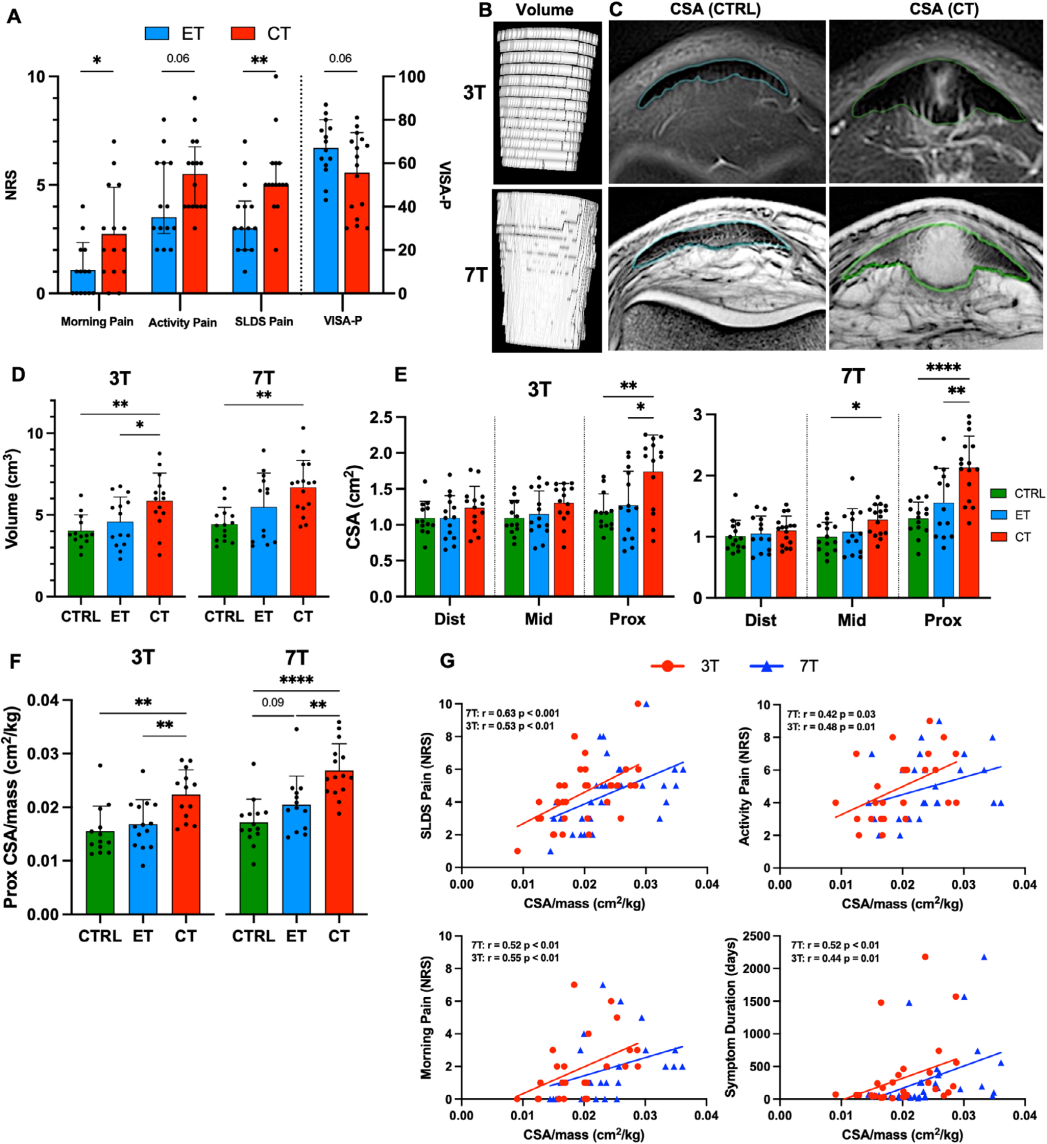
Tendon symptoms and MRI dimensions. A) Patient reported pain and function scores rated xwith NRS (0 no pain, 10 worst imaginable pain) and VISA‐P (100‐best function, 0 worst function) for early (ET) and chronic (CT) tendinopathy. B) Example of 3D patellar tendon volume computations created from 3T and 7T MRI scans. C) Examples of proximal CSA measurements of the patellar tendon performed on the same two participants with either 3T or 7T MRI scans. The measured tendon region is outlined. D) Patellar tendon volumes and E) distal, mid and proximal CSA of CTRL (healthy), ET or CT measured using 3T and 7T MRI. F) Group comparison of proximal tendon CSA related to individuals body mass. Group comparisons in (D) (E) and (F) include only one tendon per participant. G) Correlations of pain scores and symptom duration with both 3T and 7T MRI proximal patellar tendon CSA/body mass including ET and CT participants. For SLDS‐NRS and symptom duration n = 32 since both tendons were included for participants with bilateral tendinopathy, while for activity pain and morning pain *n* = 25 ‐ 26 since these were only reported for the most symptomatic tendon. Data shown as individual datapoints with mean ± SD or median and IQR for data with normal and non‐normal distribution, respectively. Unpaired *t*‐test or Mann–Whitney test performed in (A). One‐way ANOVA with Holm–Šídák correction performed in (D–F). Correlations tested using Spearman correlation coefficient in G). * *p* < 0.05, ** *p* < 0.01, **** *P* < 0.0001 NRS, numeric rating scale; SLDS, single‐leg decline squat; CSA, cross sectional area.

We included male and female patients with early (ET) or chronic (CT) tendinopathy and healthy control subjects with no history of tendinopathy (CTRL). The age range across all groups was 21–43 years, and the mean age and BMI were not significantly different between the groups (**Table** **1**). Symptom duration ranged from 25 to 3670 days across the two patient groups, and 21 patients displayed unilateral symptoms while 8 patients displayed bilateral symptoms. On the initial US screening, patellar tendon thickness was estimated and was significantly different between the three groups (Table 1). Regarding patient‐reported symptoms, both retrospective questionnaire‐based symptoms (morning pain, activity pain, and Victorian Institute of Sports Assessment – Patella (VISA‐P)) and the current pain assessed during a single leg decline squat test (SLDS pain) at screening were worse in the CT group, compared to the ET group (**Figure** **1**A).

**Table 1:** Participant characteristics. Pre‐activity: total weekly training volume loading the patellar tendon before injury. Current activity: total weekly training volume loading the patellar tendon at the time of inclusion. PT US thickness: patellar tendon thickness measured during ultrasound screening. Data are presented as mean ± SD if normally distributed or as median (range) if not. P values represent unpaired *t*‐test or Mann–Whitney test if two groups are compared, and one‐way ANOVA or Kruskal–Wallis test if three groups are compared. N/A: not applicable.

	Healthy control CTRL	Early tendinopathy ET	Chronic tendinopathy CT	P value
Sex, male/female, n	10/5	8/6	12/4	N/A
Age, years, median (range)	26.7 (21–40)	29.8 (21–43)	25.5 (21–40)	0.093
Body mass index, kg/m^2^, mean ± SD	23.8 ± 2.7	23.7 ± 2.1	24.3 ± 1.9	0.739
Unilateral/Bilateral symptoms, n	N/A	12/2	9/7	N/A
Symptom duration, days, median (range)	N/A	54 (25–71)	255 (102–3670)	<0.0001
Pre‐activity, h/week, median (range)	N/A	4.5 (1–18)	4.5 (0–14)	0.992
Current activity, h/week, median (range)	3.0 (2–12)	2.5 (0–15)	2.0 (0–11)	0.062
PT US thickness, mm, mean ± SD	4.57 ± 0.66	5.56 ± 1.76	7.97 ± 1.58	<0.0001

### 2.2 Increased MRI Pixel Intensity and Tendon Swelling in Tendinopathy

Using 3T MRI, we found that patellar tendon volume and proximal cross‐sectional area (CSA) were similar in CTRL and ET, but significantly higher in CT (Figure 1D,E). CSA was not different between groups at the mid or distal tendon region. Tendon dimensions were further studied using 7T MRI to potentially reveal more discrete tissue changes. Unlike 3T MRI, which showed swelling only in the proximal tendon in CT, 7T MRI revealed swelling also at the mid region (Figure 1E). There was considerable within‐group variation in tendon volume and CSA, especially in the tendinopathy groups. Normalizing tendon size to body dimension is recommended to avoid scaling artefacts,^[^ ^27^ ^]^ and we found that proximal CSA of healthy tendons correlated with body mass (Figure S1A, Supporting Information). After normalization (CSA/mass), significant tendon swelling was still observed in CT, and 7T MRI indicated swelling also in ET compared to CTRL (*p* = 0.09) (Figure 1F). Group comparisons were additionally tested with a higher sample size by including the contralateral tendon in healthy and bilateral tendinopathy cases. These comparisons confirmed our previous findings and further indicated that tendon size was increased in ET compared to CTRL for both volume (*p* = 0.06) and proximal CSA/mass (*p* = 0.03) (figure S2A,B, Supporting Information). Overall, 7T MRI showed slightly larger tendon dimensions and greater between‐group differences than 3T MRI, and only 7T MRI detected differences between CTRL and ET

Since it is unknown if tendinopathy affects the tendons systemically or only locally, we compared the asymptomatic supposedly healthy contralateral tendons of participants with unilateral early (Asymp ET) and chronic (Asymp CT) tendinopathy to healthy controls (CTRL). 7T, but not 3T MRI, showed a borderline higher tendon volume in Asymp CT compared to CTRL (*p* = 0.07), although proximal CSA and CSA/mass were not different between the groups (p > 0.05) (Figure S1B,C, Supporting Information).

3T and 7T MRI analysis showed that the mean pixel signal intensity was highest in the proximal tendon (Figure S1D, Supporting Information), the main site of swelling and symptoms in tendinopathy, indicating increased signal intensity may mark pathologic tissue. Further, proximal pixel intensity was clearly increased in the CT group, compared to CTRL, for both 3T and 7T MRI. This was most evident in 7T images, which also demonstrated higher pixel intensity in CT at the mid‐tendon region. However, neither 3T MRI nor 7T MRI detected differences in pixel intensity between CTRL and ET.

Both 3T and 7T MRI showed significant correlations between tendon size (proximal CSA/mass) and pain measures (Figure 1G and Figure S1E, Supporting Information). Mean proximal pixel intensity also correlated with several pain measures (Figure S1F, Supporting Information), further linking pixel intensity to tendinopathy pathology. Lastly, tendon size, but not pixel intensity, was positively correlated with symptom duration for both 3T and 7T MRI (Figure 1G and Figure S1F, Supporting Information).

### 2.3 Tendon Blood Flow is Increased and Correlates with Tendon Pain

**Figure 2:**
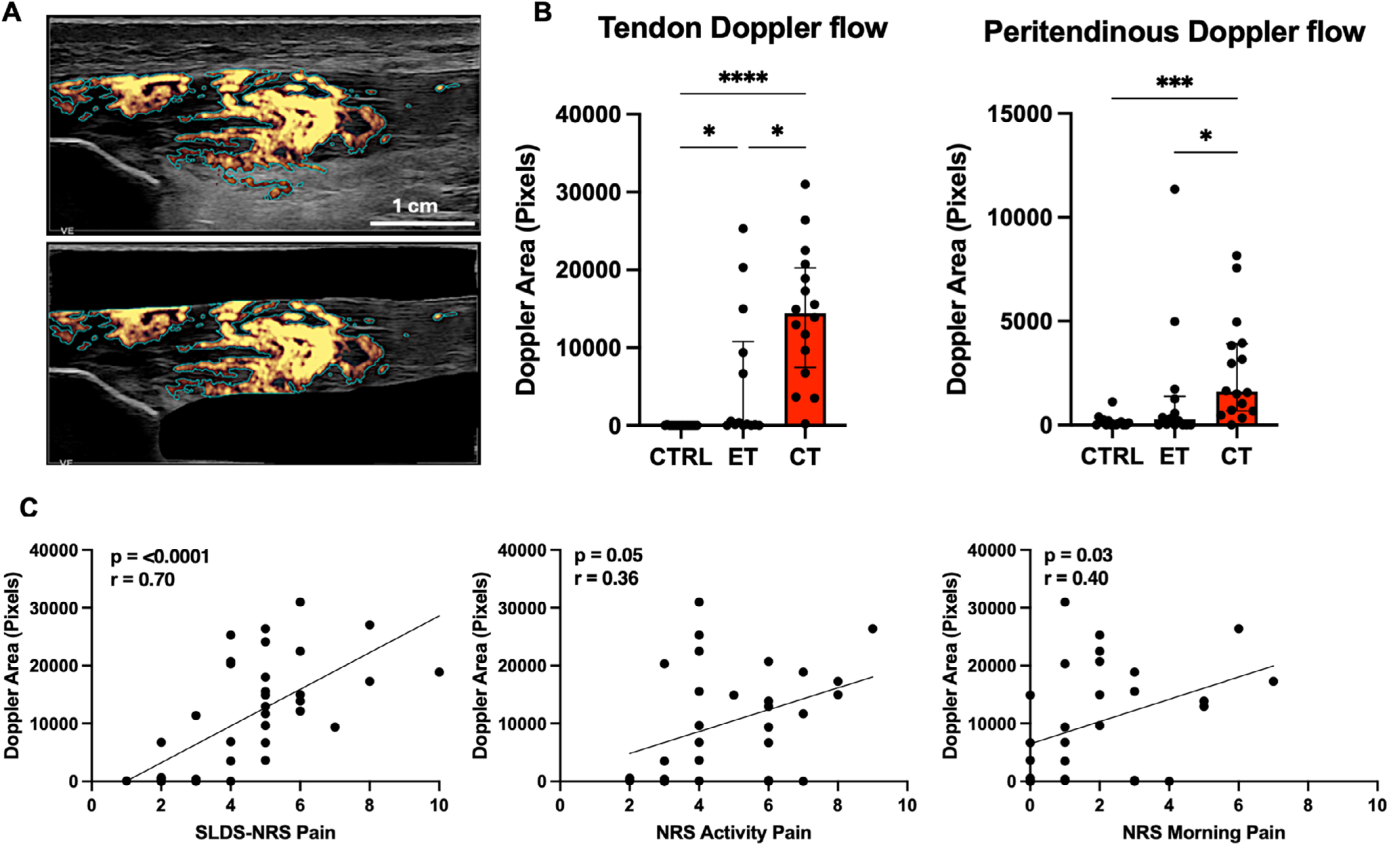
Tendon Doppler flow. A) Example of patellar tendon Doppler flow quantification of entire image (top panel) and within tendon only (bottom panel). Peritendinous Doppler flow was calculated by subtracting Doppler area within tendon from total Doppler area. B) Tendon and peritendinous Doppler flow for healthy (CTRL), early (ET) and chronically (CT) tendinopathic tendons. Group comparisons in (B) include only one tendon per participant. C) Correlations of pain scores with tendon Doppler flow including ET and CT participants. For SLDS‐NRS *n* = 37 since both tendons were included for participants with bilateral tendinopathy, while for activity pain and morning pain *n* = 29 ‐ 30 since these were only reported for the most symptomatic tendon. Data shown as individual datapoints with median and IQR. Kruskal–Wallis with Dunn's multiple comparisons test performed in (B). Correlations tested using Spearman correlation coefficient in (C). * *P* < 0.05 *** *P* < 0.001 **** *P* < 0.0001. NRS, numeric rating scale; SLDS, single‐leg decline squat.

Ultrasound power‐Doppler showed significantly increased patellar tendon blood flow in both ET and CT versus CTRL, and a further increase from ET to CT (**Figure** **2**A,B). This indicates that blood flow increases early and rises further with ongoing tendinopathy. Peritendinous blood flow increased in CT compared to CTRL and ET. As with tendon dimensions, we tested if tendon blood flow correlated with patient symptoms, analyzing only the symptomatic tendons. Tendon blood flow correlated significantly with SLDS pain, morning pain, and symptom duration, was borderline for activity pain, and was not significant for VISA‐P (Figure 2C and Figure S3A, Supporting Information).

To determine if increased blood flow was specific to symptomatic tendons, we compared Doppler flow between symptomatic and non‐symptomatic tendons in subjects with unilateral tendinopathy (ET and CT). Blood flow differed significantly between symptomatic and non‐symptomatic tendons (*p* < 0.001, 2‐way ANOVA), with a significant group x leg interaction (*p* = 0.01, 2‐way ANOVA). Subsequent multiple comparisons revealed increased blood flow in symptomatic versus non‐symptomatic tendons only in CT, not ET (Figure S3B, Supporting Information). For peritendon blood flow, there was only a main effect of the leg variable (*p* = 0.01, 2‐way ANOVA), but comparisons between the symptomatic and non‐symptomatic showed no significant differences. Thus, increased blood flow mainly occurs in the symptomatic tendons and is most pronounced in the chronic phase of tendinopathy.

### 2.4 Expansion of Tendon Vasculature in Tendinopathy

**Figure 3:**
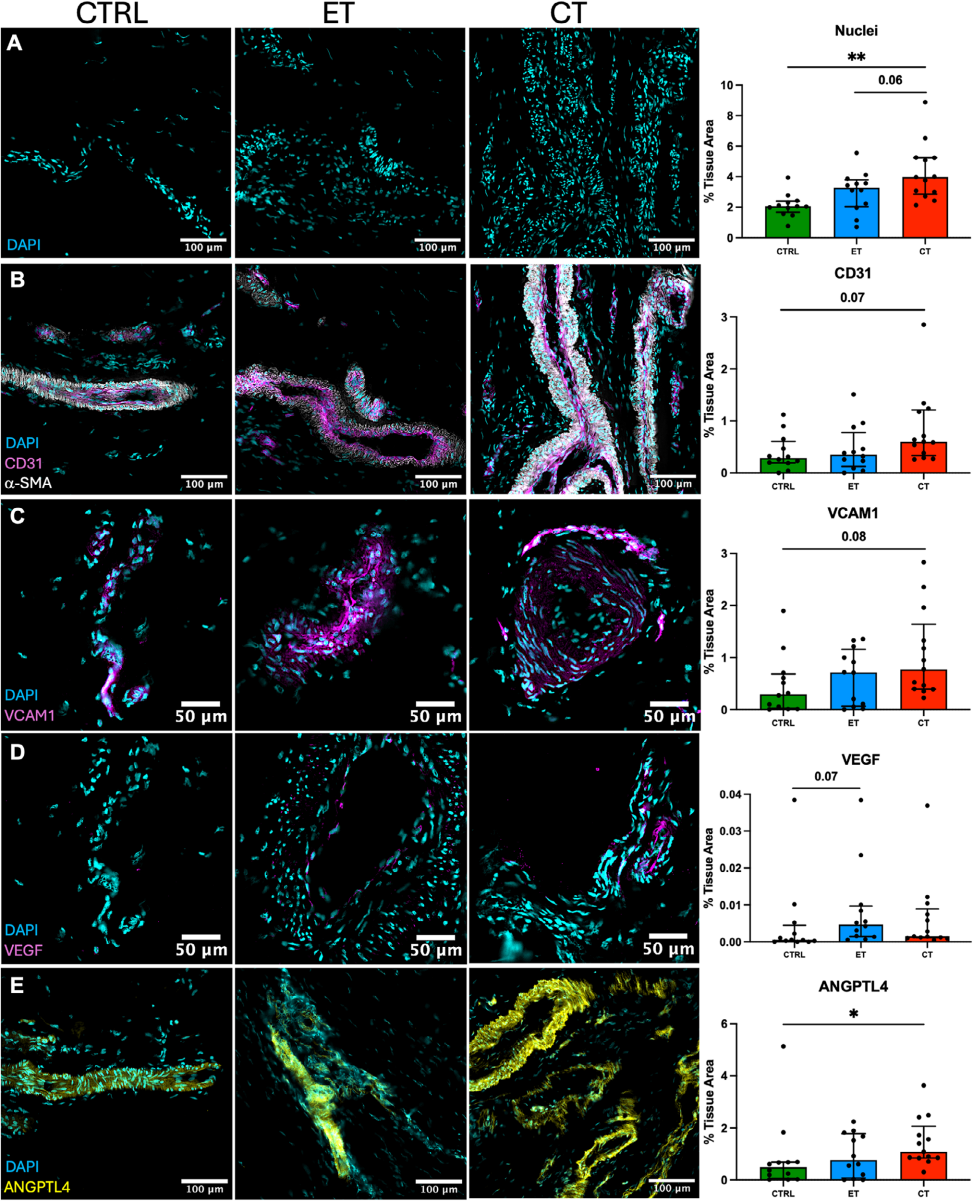
Vasculature in healthy and tendinopathic tendons. Immunofluorescent staining and quantification of A) cell nuclei (DAPI) and vascular proteins including B) CD31, C) VCAM1, D) VEGF and E) ANGPTL4 in healthy (CTRL), early (ET) and chronically (CT) tendinopathic tendons. Group comparisons include only one tendon per participant. Data shown as individual datapoints with median and IQR. P values represent Kruskal–Wallis with Dunn's multiple comparisons test. *P < 0.05, **P < 0.01.

Immunofluorescent staining was performed on patellar tendon biopsies to obtain quantitative and spatial information on tissue structures and proteins relating to vasculature, inflammation, proteoglycans, and cell type. The quantity of cell nuclei was significantly different between the groups (*p* < 0.01, one‐way ANOVA), with significantly more cells in the tendinopathic tissue samples compared to CTRL (**Figure** **3**A). Large vessels with thick α‐SMA^+^ vessel walls and smaller vessels with no apparent vessel wall (likely small arteries and capillaries) were seen in both healthy and tendinopathic samples. While vessels were located both between fascicles and within the tissue matrix in all groups, vessels within the tissue matrix were more pronounced in the tendinopathic samples. Quantitative analysis of positive staining area for vascular proteins showed significant group differences for ANGPTL4 (angiogenic protein angiopoietin‐like 4, *p* < 0.05) and a similar trend for the remaining vascular markers CD31 (endothelial cells, *p* = 0.06), VCAM1 (inflammatory‐activated endothelial cells, *p* = 0.09), and VEGF (vascular endothelial growth factor, *p* = 0.06) (p values from one‐way ANOVA), suggesting a greater vascular network in tendinopathy (Figure 3 and Figure S4, Supporting Information). Post hoc group comparisons are presented in figure 3. No significant differences were found for markers of vascular smooth muscle cells/pericytes (α‐SMA, NOTCH3, CD146, *p* = 0.22, 0.14, 0.17), fibroblasts, (CD90, *p* = 0.23), immune cells (TLR4, IL1R, CD163, *p* = 0.27, 0.28, 0.37) or proteoglycan 4 (PRG4/lubricin, *p* = 0.27) (Figure S4D, Supporting Information). Additional group comparisons, including also bilateral symptomatic tendon samples, corroborated that cellularity and vascular proteins (CD31, VCAM1, and ANGPTL4) were increased in CT and showed that NOTCH3 was significantly increased in CT versus CTRL (Figure S5, Supporting Information).

Microscopy‐based quantification of vascularity (CD31) showed a significant correlation with tendon blood flow measured by US Doppler (including both tendons in bilateral cases), confirming that microscopic biopsy vascularity reflects the measured macroscopic tissue perfusion, even though biopsies sample only a small portion of the tendon (Figure S6A, Supporting Information). Correlations were significant both if asymptomatic tendons were included (*r* = 0.47, *p* < 0.01), or excluded (*r* = 0.34, *p* < 0.01).

### 2.5 Vascular Adaptations in Asymptomatic Tendons in Patients with Tendinopathy

Since Doppler flow was found in some healthy tendons in Asymp ET and Asymp CT, we examined if they were microscopically different from healthy tendons in CTRL. Initial ANOVA testing showed no differences in vascular markers between the three groups. However, comparing the combined asymptomatic tendons in ET and CT (Asymp ET+CT) to CTRL showed that, irrespective of symptom duration, VCAM1 staining was significantly higher in Asymp ET+CT (*p* = 0.04), while CD31, VEGF, and cell nuclei were slightly but not significantly elevated (*p* = 0.19, 0.15, 0.21) (Figure S6B, Supporting Information). No other markers were significantly changed.

### 2.6 Distinct Blood Vessel Phenotypes in Tendinopathy

**Figure 4:**
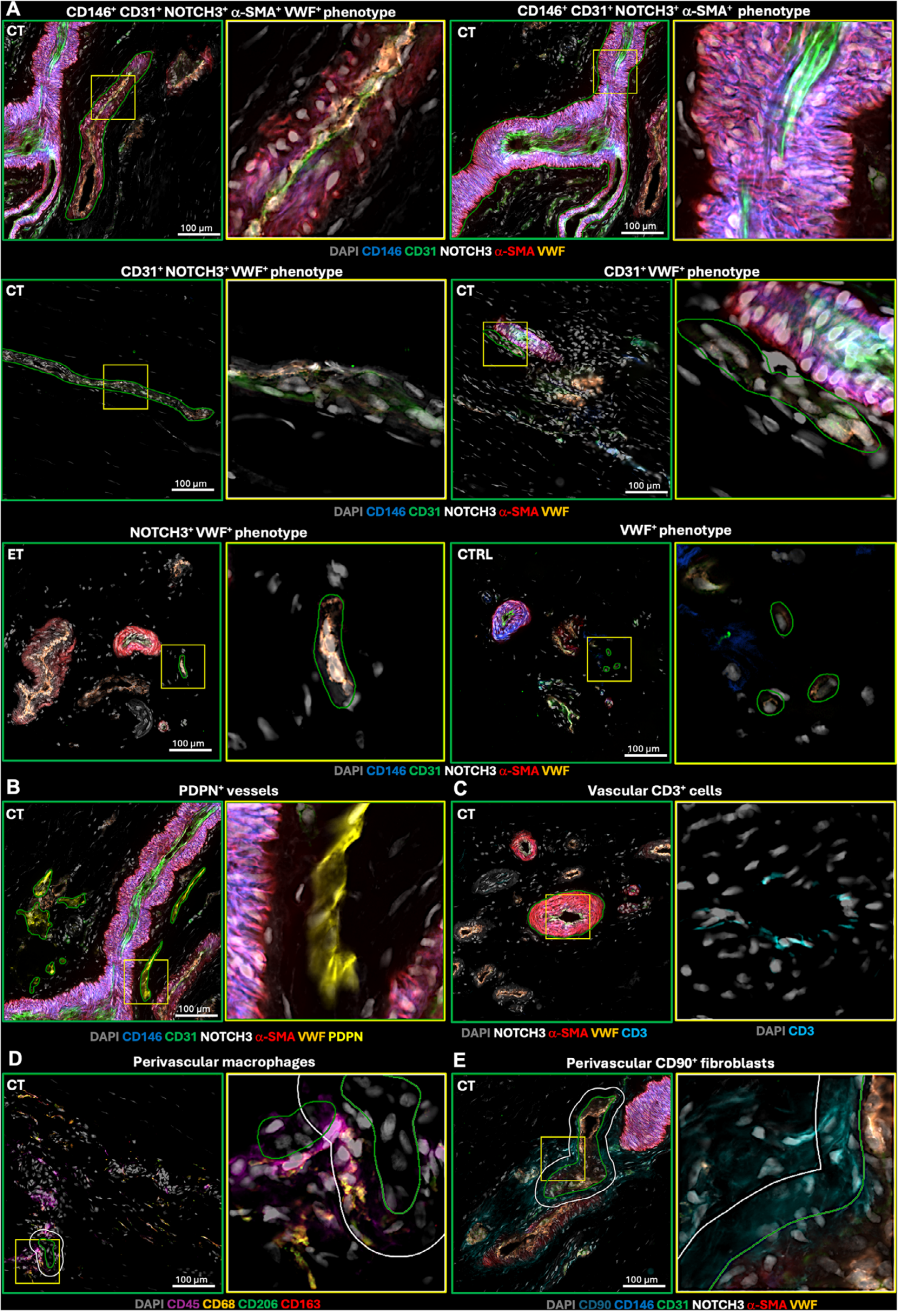
Blood vessel phenotypes in tendon. A) Examples of the most abundant vessel phenotypes observed in patellar tendon biopsies. If several phenotypes are present the annotated phenotype is indicated by green outline. Yellow box indicates the region magnified in the following image. B) Podoplanin (PDPN)+ vessels are outlined in green. C) Vascular CD3+ cells inside vessel segment (green). D) Cells positive for various macrophage markers observed in the perivascular region (white) outlined outside vessel segments (green). E) CD90+ fibroblasts observed in the perivascular region (white) outlined outside vessel segment (green). Participant group is indicated in upper left corner of images. Images analyzed using software QuPath (v.0.5.1).

**Figure 5:**
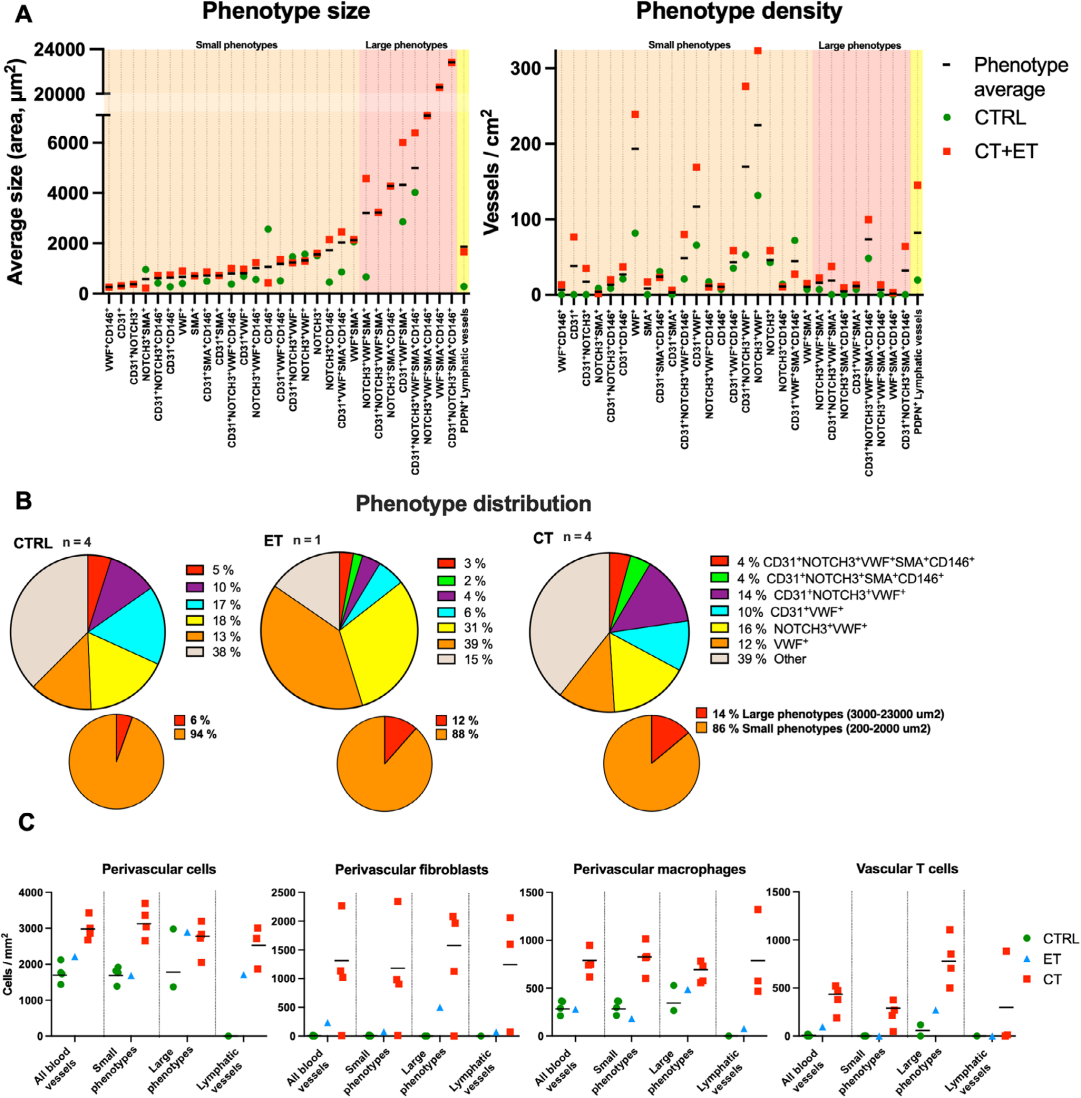
Vessel phenotype analysis. A) Average vessel phenotype size and density is shown for all samples (phenotype average) and specifically for healthy (CTRL) or tendinopathic (CT+ET) tendons. Horizontal line indicates the average of the vessel phenotype across all samples, including the asymptomatic contralateral sample in ET. B) Blood vessel phenotype distribution of small and large sized phenotypes and a selection of the most abundant phenotypes across healthy (CTRL), early (ET) and chronically (CT) tendinopathic tendons. C) Perivascular cell (total cell: DAPI+; fibroblast: CD90*; macrophage: CD68+, CD163+ and/or CD206+) and vascular T cell (CD3+, CD3+CD31+ and CD3+CD45+) quantification for all blood vessels, small phenotypes, large phenotypes and PDPN+ lymphatic vessels in CTRL, ET and CT tendon samples. Horizontal line indicates the group average. Sample n = 4 (2 bilateral samples from 2 participants) for both CTRL and CT, and n = 1 for ET. Datapoints only plotted in graph if phenotype observed in sample. No statistical testing was performed due to the low number of samples.

Using Cell DIVE imaging on a subset of samples, we observed various blood vessel subtypes in the patellar tendon sections (**Figure** **4**A). The largest and most abundant vessel phenotypes are highlighted in Figure 4 and Table S1 (Supporting Information). Lower magnification images for all groups are shown in Figure S7 (Supporting Information). We imaged vasculature‐related proteins (CD31, VWF, NOTCH3, α‐SMA and CD146) demonstrating that in both healthy and tendinopathic tendons, the largest vessels had the most complex phenotypes, expressing most markers, particularly α‐SMA^+^ vessel walls (Figure 4A). Simpler phenotypes expressing only a few vascular markers, particularly those expressing VWF, were smaller and more abundant (**Figure** **5**A and Table S1, Supporting Information). Although the sample size precluded statistical testing, average vessel size across all phenotypes appeared larger in tendinopathic than healthy samples, especially among large/complex phenotypes, while smaller/simpler phenotypes displayed similar size between groups. Vessel density was also higher in tendinopathic samples, mainly for large phenotypes, meaning small vessels comprised a relatively smaller proportion of the overall vasculature in tendinopathic tissue (Figure 5B). Certain large vessel phenotypes, including CD146^+^ CD31^+^ NOTCH3^+^ α‐SMA^+^ and CD146^+^ CD31^+^ NOTCH3^+^ VWF^+^ vessels, were only observed in ET and CT samples, suggesting certain phenotypes may potentially be specific to tendinopathy.

### 2.7 Perivascular Cell Recruitment and Expansion of Potential Lymphatic Vessels in Tendinopathy

We observed various cell types, including macrophages and CD90^+^ fibroblasts, residing in the perivascular regions of the analyzed blood vessels (Figure 4D,E). The density of perivascular cells (CD90^+^ fibroblasts, macrophages, and non‐specific cells) was not apparently different between vessel phenotypes but appeared markedly higher in tendinopathic samples compared to controls for both large and small vessel phenotypes (Figure 5C and Table S1, Supporting Information). Particularly, CD90^+^ fibroblasts were abundant in the perivascular space in the tendinopathic samples but were lacking in most of the healthy samples (Table S1, Supporting Information).

Utilizing the established T cell marker CD3, Cell DIVE analysis demonstrated various CD3^+^ cell phenotypes. We observed CD3^+^, CD3^+^CD31^+,^ and CD3^+^ CD45^+^ cells within the vessel lumen or wall (Figure 4C), and in tissue matrix cell clusters, suggesting these could be circulating or activated T cells within immune cell infiltrates.^[^ ^28^ ^]^ Next, we explored if tendinopathy influences T cell recruitment by quantifying T cells (CD3^+^, CD3^+^CD31^+,^ and CD3^+^CD45^+^ cells) observed inside the vasculature. Vascular T cells were particularly evident in the large vessel phenotypes and more abundant in tendinopathic than in healthy tissue (Figure 5C), implying activation of the adaptive immune system in tendinopathy. Vascular T cells were markedly abundant in CD146^+^ vessels (Table S1, Supporting Information). Since T cells are transported by both the cardiovascular and lymphatic systems, we investigated if T cells were also present in lymphatic vessels. We observed vessel‐like cell structures expressing the lymphatic marker podoplanin (PDPN) while weakly expressing the endothelial cell marker CD31 (Figure 4B). These potential lymphatic PDPN^+^ vessels displayed large variation in size and density between samples but were mainly found in tendinopathic samples and only scarcely in healthy samples (Figure 5A and Table S1, Supporting Information). In a few cases, T cells colocalized with PDPN^+^ vessels, possibly representing T cells circulating in the lymphatic system.

### 2.8 Proteomic Profile of Tendinopathy

**Figure 6:**
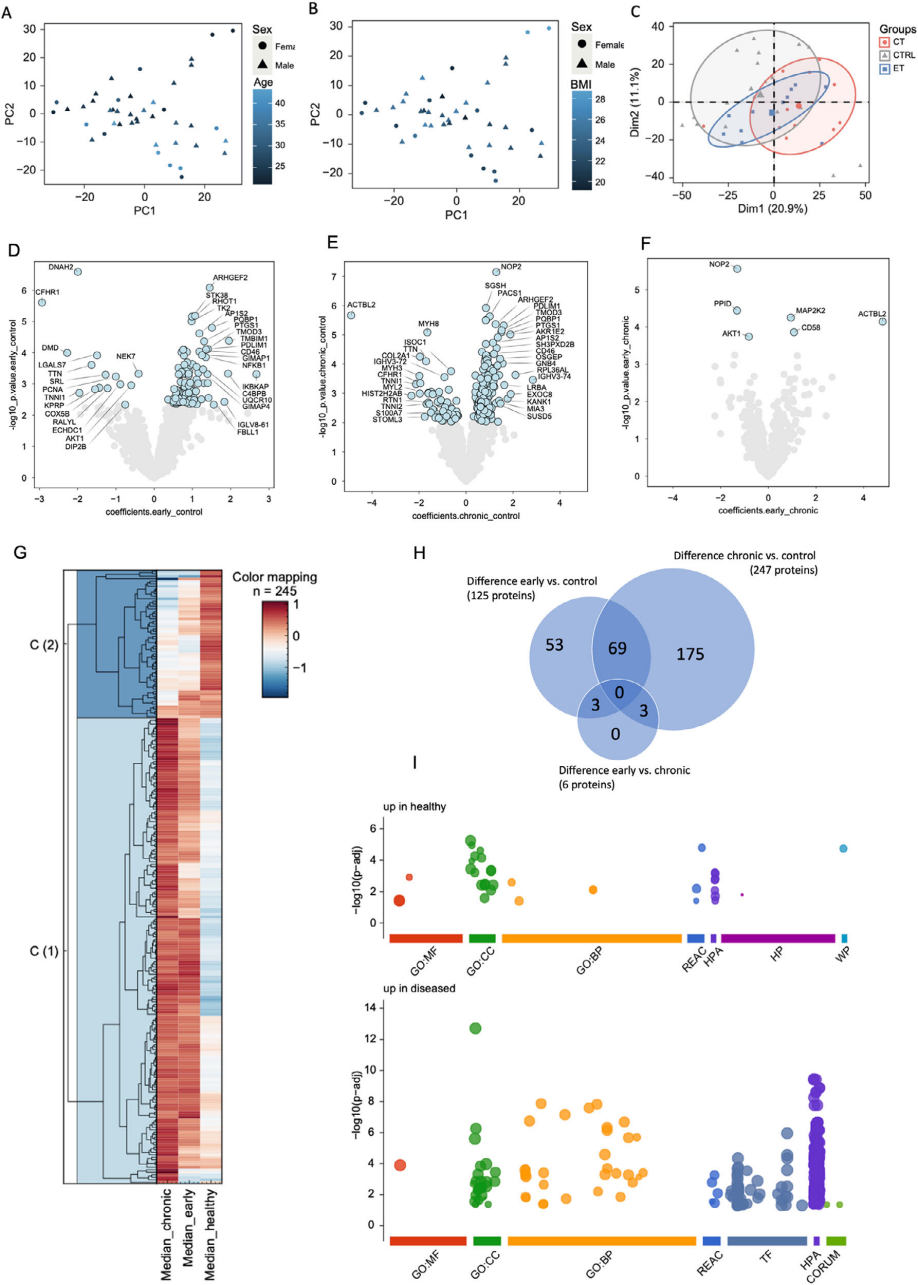
Proteomic characterization of healthy and tendinopathic tendons. Principal component analysis of A) sex and age, B) BMI and C) participant group. Negative and positive values in volcano plots indicate significantly down‐ and upregulated proteins, respectively, in D) early tendinopathy (ET) compared to healthy controls (CTRL), E) chronic tendinopathy (CT) compared to CTRL and F) ET compared to CT. G) Hierarchical clustering of the proteome comparing CTRL, ET, and CT separates the proteome in two primary clusters being C(1) upregulated in disease (ET and CT) and C(2) upregulated in healthy. H) Venn diagram showing the overlap of the significantly regulated proteins in (D), (E) and (F). I) GOterm enrichment analysis with g:profiler showed proteins upregulated in healthy (CTRL) and in disease (ET and CT). Only the most symptomatic tendon was included in the analysis for ET (n = 11) and CT (n = 12), while both the healthy tendons were included for CTRL (n = 20). Proteomic data were filtered (*q* < 0.01, Lib.*Q*.value < 0.01, PG.*Q*.value < 0.01, Global.*Q*.value < 0.01, Quantity. Quality >  0.7, Fragment.count ≥ 4) and missing values were imputed using a random forest algorithm for data with 70% data completeness. Sex, BMI, and age effects were regressed out using linear models for microarray data (LIMMA). Differential analysis was performed with ANOVA or Welch's t‐test (FDR < 0.05, 500 randomizations). GO term analysis was performed on significantly changed proteins. Gene ontology molecular function (GO:MF), gene ontology cellular component (GO:CC), gene ontology biological process (GO:BP), reactome (REAC), human protein atlas (HPA), human phenotype ontology (HP), wiki pathways (WP), transcription factor binding site (TF), protein complexes (CORUM).

Proteome analysis using liquid chromatography tandem mass spectrometry (LC‐MS/MS) identified 2615 proteins across CTRL, ET, and CT samples. Principal component analyses (PCA) revealed no segregation of the data due to sex, age, nor BMI (**Figure** **6**A,B). The most pronounced segregation was seen between CT and CTRL, whereas ET showed considerable overlap with both groups (Figure 6C). Of the 2615 proteins, significantly different abundance was found of 125 proteins for ET versus CTRL, 247 proteins for CT versus CTRL, and 6 proteins for ET versus CT (Figure 6D–F and data file S1, Supporting Information), while 69 proteins differed for both ET versus CTRL and CT versus CTRL (Figure 6H). Hierarchical clustering of the proteome showed two main clusters: cluster 1 (C(1)) with proteins more abundant in ET and CT, and cluster 2 (C(2)) with proteins more abundant in CTRL (Figure 6G). The proteomic profiles of ET and CT showed no clear cluster separation, indicating that the greatest difference in proteome was between CTRL and the two tendinopathy groups (Figure 6G,H). Gene Ontology (GO) enrichment analysis was performed on proteins with higher abundance in healthy samples and those with higher abundance in disease, respectively (Figure 6I and data file S2, Supporting Information). This revealed that cellular components (GO:CC), including the term collagen‐containing extracellular matrix, were among the most enriched terms in healthy samples. In the diseased samples, a greater number of biological processes (GO:BP) terms were enriched, including protein transport, localization, and binding, cellular localization, intracellular transport, and autophagy. This suggests that components of the extracellular matrix might be affected in tendinopathy, and that tendinopathy is not merely a degenerative process, but also involves active biologic processes. This is further supported by specific regulation of inflammation‐ and angiogenesis‐related proteins NFKB1, PTGS1(COX1), PTGES2, CD58, APOE, PDGFRα, NRTK2, VEZF1, and aligns with the observed tendon swelling and vascular adaptations. Specific investigation of vascular proteins using the GO terms “angiogenesis” (GO:0 001525), “vasculogenesis” (GO:0 001570) and “vasculature development” (GO:0 001944) revealed that protein abundance was significantly different for 5 vasculature‐related proteins between CTRL and ET, 11 proteins between CTRL and CT, and 1 protein between ET and CT, supporting that vascular adaptations are most pronounced in CT (**Table** **2**).

**Table 2:** Classification of vascular proteins by GO terms “angiogenesis” (GO:0001525), Vasculogenesis” (GO:0001570), “vasculature development” (GO:0001944) and matrix proteins by MatrisomeDB 2.0 on all detected proteins and proteins significantly different between chronic vs. control, early vs. control, and chronic vs. early. Shown as number of proteins and specific gene and protein name. Coefficients indicate group differences with higher values indicating higher protein abundance in the group first mentioned. GO, gene ontology; ECM, extracellular matrix; Control, healthy controls (CTRL); Early, early tendinopathy (ET); Chronic, chronic tendinopathy (CT).

	All proteins	Chronic vs. Control			Early vs. Control			Chronic vs. Early		
Total proteins detected	2615	247			125			6		
Vascular proteins Combined GO terms “vasculature development” (GO:0001944), “angiogenesis” (GO:0001525) and “vasculogenesis” (GO:0001570) excluding duplicates	115	11			5			1		
		Gene name	Protein name	Coefficients	Gene name	Protein name	Coefficients	Gene name	Protein name	Coefficients
		APOE	Apolipoprotein E	−0.98	PDGFRA	Platelet‐derived growth factor receptor alpha	0.70	AKT1	RAC‐alpha serine/threonine‐protein kinase	−0.82
		RASA1	Ras GTPase‐activating protein 1	0.96	AKT1	RAC‐alpha serine/threonine‐protein kinase	−0.60			
		CTGF	Connective tissue growth factor	0.96	CRKL	Crk‐like protein	0.85			
		S100A7	Protein S100‐A7	−1.49	NTRK2	BDNF/NT‐3 growth factors receptor	0.66			
		LIPA	Lysosomal acid lipase/cholesteryl ester hydrolase	0.90	VEZF1	Vascular endothelial zinc finger 1	0.80			
		PTK2	Focal adhesion kinase 1	0.72						
		NTRK2	BDNF/NT‐3 growth factors receptor	0.85			
		MIA3	Melanoma inhibitory activity protein 3	1.47			
		VEZF1	Vascular endothelial zinc finger 1	0.76			
		SFRP2	Secreted frizzled‐related protein 2	−1.07			
		RIC8A	Synembryn‐A	0.65			
Total matrisome proteins	229	12			1			0		
Core matrisome total	110	3			0			0		
Collagens	23	1			0			0		
		COL2A1	Collagen alpha‐1(II) chain	−1.97						
ECM glycoproteins	70	1			0			0		
		CTGF	Connective tissue growth factor	0.96						
Proteoglycans	17	1			0			0		
		HAPLN1	Hyaluronan and proteoglycan link protein 1	−0.80						
Matrisome‐associated total	119	9			1			0		
ECM‐affiliated proteins	31	2			1			0		
		LGALS7	Galectin‐7	−1.18	LGALS7	Galectin‐7	−1.49			
		SEMA3B	Semaphorin‐3B	−0.64						
ECM regulators	64	2			0			0		
		PLOD3	Multifunctional procollagen lysine hydroxylase and glycosyltransferase LH3	0.79						
		SERPINB3	Serpin B3	−1.20						
Secreted factors	24	5			0			0		
		S100A8	Protein S100‐A8	−0.80						
		FLG	Filaggrin	1.10						
		S100A7	Protein S100‐A7 (psoriasin)	−1.49						
		SFRP4	Secreted frizzled‐related protein 4	1.08						
		SFRP2	Secreted frizzled‐related protein 2	−1.07						

### 2.9 Late Alterations of Tendon Extracellular Matrix

To further evaluate whether the proteins with different abundances were related to the extracellular matrix (ECM), the online database MatrisomeDB 2.0^[^ ^29^ ^]^ was used to identify matrix‐related proteins. Of the total 2615 proteins, 229 were identified as matrisome proteins, including 110 core matrisome proteins and 119 matrisome‐associated proteins (Table 2 and data file S3, Supporting Information). For CTRL versus CT, 3 core matrisome proteins differed significantly; one collagen, one ECM glycoprotein, and one proteoglycan, and 9 matrisome‐associated proteins; two ECM‐affiliated proteins, two ECM regulators, and. For CTRL versus ET, no core matrisome proteins and only one matrisome‐associated protein, ECM‐affiliated protein, differed. No matrisome proteins differed significantly between ET and CT. Thus, changes in tendon extracellular matrix proteins occurred primarily between the healthy and the chronic phase of tendinopathy.

### 2.10 Coupling of Observations to Clinical Improvement of Tendinopathy

To support the clinical implications of our findings, we analyzed data from a previous clinical trial involving patients with early‐onset Achilles tendinopathy (*n* = 69).^[^ ^30^ ^]^ To evaluate the potential therapeutic benefit of treating tendinopathy early, we compared clinical improvements between patient groups with varying symptom duration (**Table** **3**). We found that treating (by reducing pain‐provoking activities and performing controlled moderate strengthening exercises) tendinopathy in the very early phase (< 1 month) resulted in a quicker recovery of tendon function than if tendinopathy had lasted somewhat longer (1–3 months).

**Table 3:** **Influence of symptom duration on recovery time in human Achilles tendinopathy**. 69 patients with Achilles tendinopathy were treated with load reduction of pain‐provoking activities and substitution of controlled moderate strengthening exercises. Tendinopathy severity was evaluated with a tendon function score (VISA‐A) ranging from 0 to 100, where lower scores indicate poorer function. Data are extracted from a previous study finding no effect of treatment with anti‐inflammatory medication in tendinopathy (30). In the present manuscript patients were separated into three groups based on symptom duration. * = Significantly higher increase from baseline, compared to group with 2–3 months symptom duration. For description of statistics see previous publication (30).

Patients (no)	Duration of symptoms	VISA‐A score (0–100) Baseline	Improvement from baseline to 12 weeks	Improvement from baseline to 52 weeks
18	<1 month	67 ± 3	34 ± 3 % *	35 ± 3 %
29	1–2 months	67 ± 4	21 ± 3 %	37 ± 2 %
22	2–3 months	70 ± 3	12 ± 2 %	20 ± 5 %

## 3 Discussion

**Figure 7:**
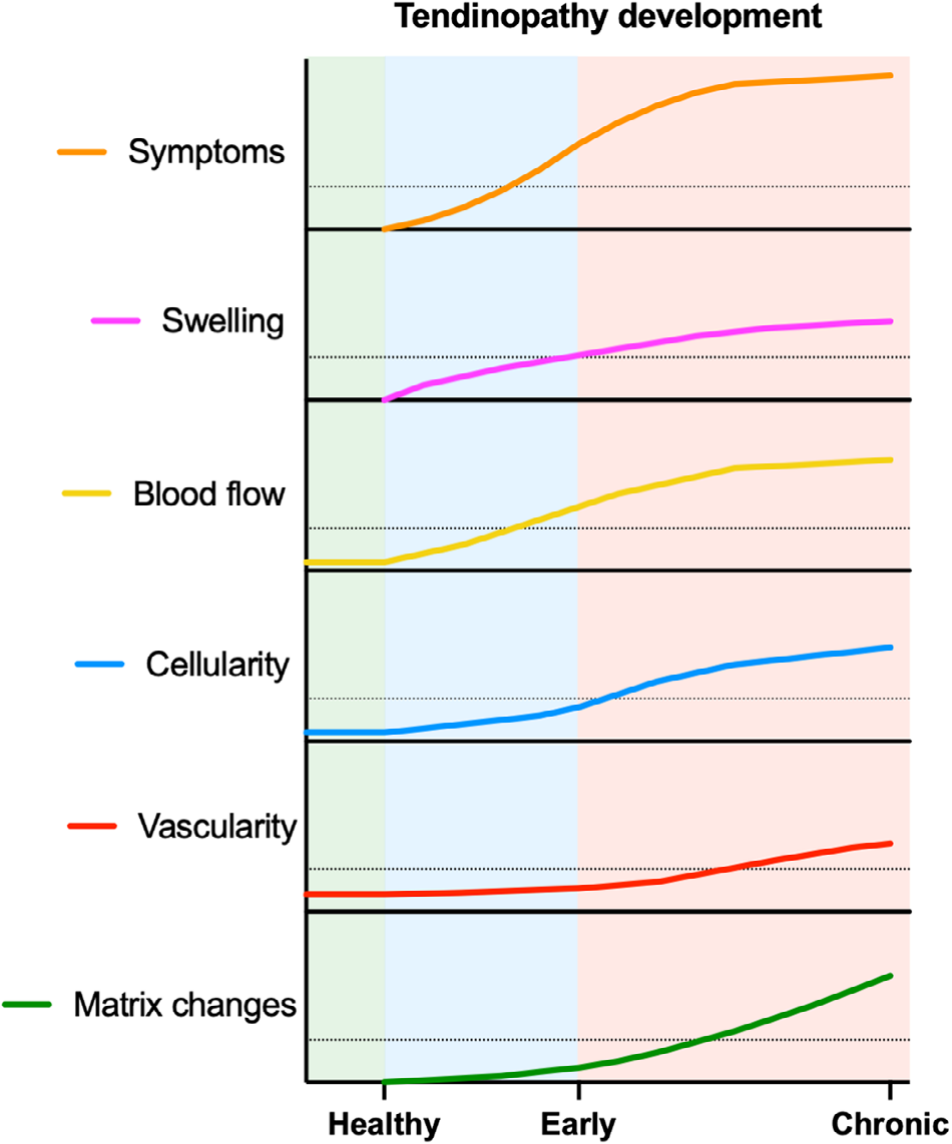
Proposed timeline of tendinopathy development. The x‐axis shows the timepoints investigated in the current study, and the y‐axis shows the different tendinopathy hallmarks investigated using various methodological approaches. The curved lines represent the hypothetical development of these hallmarks based on the cross‐sectional findings in this study. The dotted line in each plot represents a speculative threshold based on this study's findings, whereafter the development results in clinically or methodologically detectable changes.

In this study, several methodologic approaches were used to investigate the detailed pathogenesis of human patellar tendinopathy. Our data shows that the early phase of tendinopathy is dominated by pain symptoms that are associated with tendon swelling and increased blood flow, while the chronic phase is more distinctly defined by angiogenesis and protein changes in the matrisome (**Figure** **7**). Tendon blood flow gradually increased in early and chronic tendinopathy, but immunofluorescence microscopy and proteomics suggested increased vascularization, mostly in chronic tendinopathy. This divergence suggests that blood flow increases in ET due to regulation in existing vessels, rather than an expansion of the vascular network. Further increased flow in CT might then represent vascular growth, supported by our findings of increased markers of vascularity (CD31, VCAM1) and angiogenesis (ANGPTL4, VEGF). Increasing flow rate and angiogenesis may occur simultaneously during tendinopathy development, supported by our proteomics data showing upregulation in both ET and CT of VEZF1 – an endothelial cell‐specific transcription factor promoting vascular development and angiogenesis,^[^ ^31^, ^32^, ^33^ ^]^ which has not been demonstrated in tendinopathy previously. However, since increased blood flow itself stimulates angiogenesis,^[^ ^34^, ^35^ ^]^ it is likely that perfusion initially increases within existing vessels, which subsequently promotes expansion of the vasculature. We were unable to address whether the increased vasculature in CT was accompanied by nerve infiltration as previously shown.^[^ ^21^ ^]^ However, our proteomics data suggested neural tissue adaptations in chronic tendinopathy indicated by a decrease in semaphorin‐3B (SEMA3B), an inhibitor of angiogenesis and axonal guidance,^[^ ^36^, ^37^ ^]^ possibly allowing for neurovascular expansion in tendinopathy as seen in other diseases.^[^ ^38^, ^39^ ^]^

Recent studies suggest that proteoglycan (PG) and glycosaminoglycan (GAG) accumulation is important in tendinopathy pathology^[^ ^14^, ^15^, ^40^ ^]^ due to their hydrophilic properties attracting water and causing tissue swelling.^[^ ^16^ ^]^ In early tendinopathy, increased unbound water correlates and likely contributes to increased tendon size,^[^ ^7^ ^]^ and since PG and GAG production can occur within hours to days in the tendon,^[^ ^40^ ^]^ tendon swelling is likely an early indicator of tendinopathy. We previously observed signs of tendon swelling in early‐stage tendinopathy,^[^ ^7^, ^8^ ^]^ and the current study similarly showed increased normalized tendon size in ET using 7T MRI. Accumulation of GAGs and tendon swelling can increase intratendinous pressure,^[^ ^16^ ^]^ which may contribute to tendinopathy pain symptoms.^[^ ^40^ ^]^ Although we did not directly measure intratendinous pressure, the presence of swelling and its correlation with pain support the hypothesis that elevated intratendinous pressure drives pain in tendinopathy.

Studies have detected IL1R^+^, TLR4^+,^ and CD163^+^ inflammatory cells in tendinopathy,^[^ ^18^, ^19^, ^41^ ^]^ implying activation of inflammatory pathways. We found these cells in both healthy and tendinopathic samples without any difference, although some tendinopathic samples displayed markedly high CD163^+^ macrophage content. We previously observed similar inter‐subject variation of tissue macrophages in plantar fasciitis,^[^ ^15^ ^]^ suggesting inflammation may be more pronounced in some individuals. Using Cell DIVE, we observed macrophage recruitment around vessels in tendinopathic samples, suggesting that macrophage relocation, despite unchanged total abundance, may influence tendinopathy since vascular cell crosstalk can regulate blood flow^[^ ^42^ ^]^ or facilitate inflammation,^[^ ^43^, ^44^, ^45^ ^]^ angiogenesis^[^ ^46^, ^47^ ^]^ and fibrosis.^[^ ^48^ ^]^ Supporting this, proteomic data of both ET and CT samples showed a significant increase in the inflammatory transcription factor NF‐κB, which has been implicated in tendinopathy^[^ ^18^, ^19^, ^49^ ^]^ and could, through its involvement in macrophage maturation and differentiation, contribute to perivascular recruitment of macrophages. Prostaglandins and prostaglandin synthases, which are also regulated in tendinopathy,^[^ ^18^, ^19^, ^41^, ^50^, ^51^ ^]^ mediate inflammation through the NF‐κB signaling pathway^[^ ^52^, ^53^, ^54^, ^55^ ^]^ and regulate tendon blood flow.^[^ ^56^ ^]^ We found upregulation of prostaglandin synthases PTGS1 (COX‐1) and PTGES2 in both ET and CT, further supporting activation of inflammatory pathways and blood flow regulation in tendinopathy.

CD90^+^ fibroblasts are suggested to mediate tendinopathy hallmarks, including hypercellularity, structural changes, and vascular response.^[^ ^57^, ^58^ ^]^ We did not observe more CD90^+^ fibroblasts in tendinopathic samples, but Cell DIVE showed pronounced accumulation of these cells to the perivascular regions. This may facilitate pathological fibroblast–vascular cell crosstalk, as seen in rheumatoid arthritis, where endothelial NOTCH3 signaling in perivascular CD90^+^ fibroblasts contributes to disease progression.^[^ ^59^, ^60^ ^]^ Combined, these findings suggest that inflammation‐related signaling, including NF‐κB, is present in both early and chronic tendinopathy, and although total abundance may not change, perivascular recruitment of macrophages and CD90^+^ fibroblasts may drive pathological features in tendinopathy.

In healthy tendons, lymphatic vessels are limited to surrounding tissue,^[^ ^61^, ^62^ ^]^ but we observed numerous potential lymphatic PDPN^+^ vessels alongside blood vessels in the core tissue of tendinopathic tendons. These appeared to coincide with immune cells, particularly T cells, suggesting a link between vascular infiltration and adaptive immune activation in tendinopathy.^[^ ^61^ ^]^ Interestingly, several phenotypes of CD3^+^ T cells were observed, locating both inside blood and PDPN^+^ vessels and within the tissue matrix. Besides CD3^+^ cells, CD3^+^CD31^+^ cells are located inside vessel lumens and within vessel walls. CD31 is not a common T cell marker, but during early vessel development, blood‐derived “angiogenic” CD3^+^CD31^+^ T cells can migrate into ischemic tissue, contributing to endothelial cell proliferation^[^ ^63^ ^]^ and CD31^+^ cells may engulf CD3^+^ T cells to form cell‐in‐cell structures.^[^ ^64^ ^]^ Thus, the observed CD3^+^CD31^+^ cells may represent migrating T cells contributing to angiogenesis. Further, CD3^+^CD45^+^ cells located in blood vessels and tissue matrix cell clusters, possibly representing activated T cells^[^ ^28^ ^]^ recruited due to tendinopathy. Altogether, both blood vessels and potential lymphatic vessels infiltrate the tendon in human tendinopathy and may facilitate T cell involvement in tendinopathy pathology.

Group‐wise PCA of proteomics data indicated gradual proteome changes during tendinopathy progression, with distinct protein subsets regulated at different stages. A subset of 56 proteins changed significantly between CTRL and ET, but not between CTRL and CT (Figure 6H), suggesting these changes are transient and specific to early tendinopathy. An example of this pattern includes inhibitory regulation of the PKN1‐AKT1 signaling pathway, which has been implicated in tenocyte cell proliferation^[^ ^65^ ^]^ and tenogenesis of mesenchymal stem cells.^[^ ^66^ ^]^ Another proteome subset (69 proteins) was altered in ET and remained altered in CT, including NFKB1, PTGS1(COX1), PTGES2, and VEZF1, which promote inflammation and angiogenesis. These changes may reflect ongoing processes in ET and CT, such as tendon swelling and increased blood flow, which MRI and US Doppler suggest are already present in ET but become more pronounced in CT. A large part of the proteome (178 proteins), including 11 vasculature‐related proteins, was changed only between CTRL and CT. Interestingly, most of these proteins did not differ between ET and CT, suggesting they were modulated early but only reached significantly different levels later. This delayed manifestation aligns with the more pronounced changes in tendon cellularity and vascularity we observed in CT using immunofluorescence microscopy.

Matrisome analysis showed that matrix protein changes detectable with the utilized workflow appear predominantly in chronic tendinopathy. We previously investigated patients with tendinopathy symptoms for 1, 2, or 3 months and found elevated mRNA for matrix‐stimulating proteins, including CTGF, only after 2–3 months.^[^ ^8^ ^]^ In the current study, matrix proteins, including CTGF, also rose after 3 months of symptoms, suggesting fibrotic activity at the later stage.^[^ ^67^ ^]^ Our proteomic data suggested early activation of angiogenic and inflammation‐related signaling. Recent studies suggest such pathways contribute to structural alterations of the tendon matrix in tendinopathy.^[^ ^57^, ^68^ ^]^ Thus, we propose that matrix remodeling is a progressive feature that manifests in chronic tendinopathy but is primed by early angiogenic/hypoxic and possibly inflammatory signaling. Therefore, future investigations should in more detail address changes in cellular activity, proliferation, and metabolism during early tendinopathy. It should be noted that other proteomic workflows specialized for matrix proteins, such as proteoglycans, might have identified early changes not detectable with the current workflow dedicated for a broad range of proteins.

Our findings suggest that changes in cell proliferation or signaling initiate early tissue responses, including tendon swelling and increased blood flow. If persistent, these processes contribute to more substantial pathological features in chronic tendinopathy, including neovascularization and progressive extracellular matrix disruption. This sequence of events highlights a potential window for intervention targeting early tissue responses before irreversible matrix damage occurs.

It is unknown whether tendinopathy pathology is limited to the symptomatic tissue region. Tissue changes occur throughout the entire tendon following injury,^[^ ^15^, ^69^ ^]^ and unilateral tendinopathies often progress to bilateral symptoms, suggesting a systemic component to tendinopathy. A recent study suggested vascular changes may precede or even occur independently of overt tendinopathy symptoms.^[^ ^68^ ^]^ Our data indicated increased vascularization, but not swelling, in contralateral asymptomatic tendons. This could suggest that tendon swelling is more directly coupled to symptomatic tendinopathy, possibly explained by changes in intratendinous pressure, while subtle vascular changes in the asymptomatic contralateral tendons could result from prolonged exposure to systemic factors caused by hyperperfusion or angiogenic signaling at the tendinopathic region. This notion would support that systemic factors play a role in tendinopathy progression.

Our previous findings in patients with Achilles tendinopathy (Table 3) showed that shorter symptom duration is associated with faster recovery,^[^ ^30^ ^]^ supporting that more “reversible” changes occur early in tendinopathy (including vasodilation and fluid accumulation), while more “permanent” changes of matrix structures occur in the later phase, thus highlighting the importance of early intervention. Interestingly, this opens possibilities for treatments involving the manipulation of the early events in tendinopathy. Patches administering vasodilatory nitric oxide (NO) to the tendon have shown modest beneficial effects in animal models of tendon healing^[^ ^70^ ^]^ and human clinical trials of tendinopathy.^[^ ^71^ ^]^ However, only chronic tendinopathy has been studied, where marked neovascularization is already present, and it is unknown if vasodilatory drugs could be more effective if applied in the early phases of tendinopathy, potentially preventing excessive neovascularization. Furthermore, injecting hyaluronidase in human tendons acutely decreases the glycosaminoglycans (GAG) and water content, thus lowering intratendinous pressure.^[^ ^16^ ^]^ Such effects could likely mitigate or reverse tendon swelling if applied in early tendinopathy. These studies show that the early changes we describe in tendinopathy, including altered blood flow and swelling, can be clinically targeted and have shown therapeutic potential. However, as these treatments have only been tested in chronic tendinopathy, our study provides a rationale for exploring the use of these or similar treatments in early‐stage tendinopathy.

Our study has limitations. Our cross‐sectional design was well‐suited to identify differences between patients in the early and chronic stages of tendinopathy, but a prospective longitudinal study with repeated measures and tissue sampling would ideally capture within‐subject progression from acute to chronic tendinopathy. However, repeated sampling is not feasible in tendons since the biopsy procedure itself upregulates tendon cell activity and matrix protein expression, persisting up to several months, even if the second biopsy location differs from the initial one.^[^ ^72^, ^73^ ^]^ Therefore, currently, the present design optimally captures tendinopathy development in humans. Longitudinal tendinopathy animal models allow manipulation and detailed molecular description of the tendon during traumatic events, providing valuable insights. However, currently, no models truly mimic clinical symptoms of human tendinopathy,^[^ ^74^, ^75^ ^]^ limiting the translational value. Our study design supports clinically relevant insight into human tendinopathy development. However, the cross‐sectional design prohibits firm conclusions on the developmental trajectory from early‐ to chronic‐stage tendinopathy, and thus the proposed disease progression needs further validation Additionally, defining early tendinopathy by symptom duration allows potential misclassification due to recall bias, and the 90 day cutoff is based on convention which might not represent the optimal timepoint for distinguishing between early and chronic tendinopathic tissue changes. Having tendon thickening, Doppler flow, or hypoechogenicity as an inclusion criteria also risks biasing the ET group toward participants with more pronounced tissue changes. Lastly, the large symptom duration heterogeneity in CT could influence data interpretation, and the small sample size used in Cell DIVE analysis precluded statistical testing, making the results purely descriptive.

Collectively, our findings demonstrate that increases in tendon blood flow and swelling occur before vascular growth and matrix disruption, suggesting targeting of these processes in early identified patients, potentially preventing the development of chronic tissue changes. This was supported by faster clinical recovery in patients with shorter symptom duration upon initiation of tendinopathy treatment in a separate study. Our findings of perivascular cell recruitment in tendinopathy and detailed description of distinct vessel phenotypes, including potentially tendinopathy‐specific phenotypes, may guide future efforts to understand tendinopathy pathology, which is necessary to develop better treatment strategies.

## 4 Experimental Section

### Study Design

The study used a cross‐sectional design investigating tendinopathy pathology during the development of tendinopathy by including human patients with early tendinopathy, chronic tendinopathy, and healthy control subjects. Various hallmarks of tendinopathy were studied using clinically relevant non‐invasive methods, including US and MRI. Additional methods, including advanced tissue imaging approaches and proteomics requiring invasively obtaining tissue biopsies, were utilized to gain a molecular and physiological understanding of the underlying pathology during tendinopathy development. Sample size calculations could be determined from previous studies regarding chronic tendinopathy, but no suitable studies have previously performed group comparisons of healthy subjects and patients with early tendinopathy. For the various analyses, samples were randomized and analyzed in a blinded fashion.

### Study Approval and Inclusion

The study was approved by the Danish Regional Ethical Committees of the Capital Region (H‐18048248) and the Danish Data Protection Agency (VD‐2019‐150), and all participants provided informed consent prior to participation. The study was conducted at the Institute of Sports Medicine, Copenhagen, Bispebjerg Hospital, Denmark. Participants were recruited from 2019 to 2022 via advertisement on social media and were initially scheduled for a screening visit. To be included, subjects had to be sports active, between 18 and 45 years old, and have a BMI between 18.5 and 30. Further inclusion criteria for the early and chronic tendinopathy groups included symptom onset within <90 (early group) or > 90 (chronic) days at inclusion, activity related pain in the patellar tendon, palpation pain in the proximal part of the patellar tendon, and at least one of the following findings during US investigation: 1) thickening of anterior‐posterior diameter on symptomatic side, 2) increased power Doppler signal on symptomatic side, 3) hypoechoic area corresponding to the symptomatic area of tendon. These findings were confirmed by a clinician prior to inclusion. The healthy control subjects were matched with the tendinopathy patients on training volume, gender, and age as best possible. Exclusion criteria were previous surgery or injections in the knee on the affected side, smoking, known arthritis, diabetes or hypercholesterolemia, and contraindications for MRI, including ferromagnetic objects, pregnancy, or lactation. For the two tendinopathy groups, further exclusion criteria were any previous injuries to the affected patellar tendon, and finally, the early tendinopathy patients should not yet have started any systematic treatment for tendinopathy. At the initial visit, a comprehensive bilateral US investigation was performed, and all subjects completed questionnaires regarding their physical activity level, patellar tendon pain (morning pain and activity pain), and function (VISA‐P). Subjects also performed a standardized single‐leg decline squat (SLDS) test on both legs to assess current pain symptoms. A secondary visit was scheduled as soon as possible after the first visit, during which subjects underwent both 3T and 7T MRI scans, followed by bilateral percutaneous patellar tendon biopsies. For subjects with tendinopathy, a third visit was also scheduled for consultation and initiation of a rehabilitation program with a physiotherapist.

For studying clinical recovery time following tendinopathy treatment, 69 active sports participants (mean age 41 ± 2 yrs) were included, who all had activity‐related pain in the Achilles tendon. They were originally included to investigate the use of anti‐inflammatory medication (NSAID) in tendinopathy, but no effect was observed.^[^ ^30^ ^]^ All patients were treated by reducing their training load and performing controlled moderate strengthening exercises. Patients were divided into 3 groups that had symptoms for either < 1 month, 1–2 months, or 2–3 months (Table 3), and clinical improvement on VISA‐A (where 0 is the lowest possible Achilles tendon function and 100 indicates full function) was registered 12 and 52 weeks after rehabilitation.

### Ultrasound Doppler Flow Measurements

All participants underwent bilateral B‐mode and power Doppler US examinations at Bispebjerg Hospital to determine tendon thickness and blood flow, respectively. Tendon thickness was assessed while subjects were seated with 90‐degree flexion of the knee by measuring the anterior‐posterior distance at the thickest point of the tendon in the transversal plane, excluding the epitenon or paratenon. For Doppler assessment, subjects were arranged lying in the supine position, with the knees fully extended to avoid any restrictions in blood flow during measurements. When the region with the most Doppler signal was identified, a time series of scanning frames was recorded. Doppler flow was analyzed in a blinded manner and in duplicates. The software Fiji (v2.14.0) was used for automatic detection of the frame with the highest area of positive Doppler signal. The Doppler flow area was not normalized to tendon size since this also changes in tendinopathy. Instead, the absolute Doppler area was used for group comparisons. Since Doppler flow can often be present in the peritendinous area, separate quantifications were performed for tendon tissue only and for peritendon tissue only. The peritendon region was defined as all tissues surrounding the tendon, including primarily the Hoffa's fat pad and the subcutaneous tissue (Figure 2A).

### Magnetic Resonance Imaging

Axial and sagittal MRI scans were obtained using a 3T Siemens Verio scanner using a TE (echo time) of 43 ms and a TR (repetition time) of 3830 ms (image size: 384 x 384; slice thickness: 3 mm) at Bispebjerg Hospital. Axial and sagittal MRI scans were also obtained with a 7T Philips Achieva scanner using a TE of 2.7 ms and TR of 20 ms (image size: 640 x 640; slice thickness: 3 mm) at the Danish Research Center for Magnetic Resonance (DRCMR), Hvidovre Hospital. Participants were scanned in a supine position with a pillow supporting their knee to ensure a slightly flexed position. Participants were informed to refrain from strenuous physical activities on the day of scanning. Tendon dimensions were measured in an open‐source PACS system (Horos, v4.0.1, Geneva, Switzerland). The tendon was manually outlined in every image slice by a blinded investigator to obtain the 3D volumes. The most proximal and distal slices wherein the tendon was free of the bone insertion were used to define the proximal and distal tendon regions, respectively, while the mid tendon regions were defined as the middle slice between the proximal and distal slices.

### Tissue Collection

Patellar tendon biopsies were obtained under sterile conditions using a 14‐G automatic biopsy needle (Bard Magnum Biopsy Instrument; CR Bard, Covington, GA). Subjects were prone with the knees in 90° flexion. Hair was removed from the biopsy area, and the skin was anesthetized using 2 mL 1% lidocaine. The region was sterilized with 0.5% chlorhexidine alcohol, and the tendon was exposed by a 5 mm incision into the skin. For the tendinopathy patients, the target biopsy region was the most affected region guided by the previous US and MRI images, and in healthy subjects, the proximal central tendon was used as the standard target region. The obtained tendon tissue (2–20 mg wet weight) was placed on a drop of saline to avoid drying, while fat, subcutaneous, and loose connective tissue were dissected. The tissue was then embedded in Tissue Tek (Sakura Finetek) and snap frozen in isopentane pre‐cooled by liquid nitrogen and then stored at −80 °C.

### Tissue Sectioning

Frozen tissue samples were sectioned using a cryotome (−20 °C) at 10‐µm thickness in longitudinal orientation. Tissue sections were mounted on glass slides (Superfrost Plus, Thermo Fischer Scientific) for immunofluorescence microscopy or collected in a plastic tube for proteomic analysis. This was done in an alternating fashion throughout the tissue sample to ensure that analyses would, as best possible, represent the entire tissue sample and not be affected by regional differences. The tissue was then stored at −80 °C until further analyses.

### Multiplex Immunofluorescence

Sections were allowed to thaw, immersed in phosphate‐buffered saline (PBS), and bleached under LED overnight to quench autofluorescence. The next day, sections were incubated at 4 °C overnight with primary antibody mixtures (antibodies used listed in Table S2, Supporting Information) diluted in PBS with 0.1% Tween 20 and 5% normal serum. Then, sections were washed 3 times for 5 min in PBS and incubated at room temperature for 1 h with secondary antibody mixtures diluted in PBS with 0.1% Tween 20 and 5% normal serum. After incubation, sections were again washed 3 times for 5 min in PBS, and then mounted with ProLong Gold (Thermo Fisher Scientific) with 4′, 6‐diamidino‐2‐phenylindole (DAPI), and coverslipped. Slides were then sealed and stored at 4 °C until image acquisition. For negative control staining, the primary antibodies were replaced with an isotype antibody cocktail of mouse or rabbit IgG1, IgG2a, IgG2b, IgG3, and IgGM (Dako). Image acquisition was performed on a Zeiss Axioscan 7 slidescanner at 20x magnification.

### Cell DIVE

For explorative immunofluorescence analysis, a Leica Cell DIVE multiplex imaging system was used. Sections were allowed to thaw, fixed in precooled (−20 °C) 1:1 mixture of ethanol/acetone for 2 min and then in 4% paraformaldehyde (PFA) for 5 min, followed by rehydration in PBS for 15 min. Sections were then blocked in PBS with 10% donkey serum and 3% bovine serum albumin (BSA) for 1 h at room temperature and washed in PBS for 5 min. Sections were then immersed in PBS and bleached under LED overnight to quench autofluorescence. The next day, the slides were incubated with DAPI by immersion in DAPI working solution (PBS with 0.2% DAPI dilactate) for 15 min, washed in PBS for 5 min, and quickly rinsed in double‐distilled H_2_O before cover slipping. Slides were then imaged on the Cell DIVE system to determine background signal, whereafter coverslips were removed by immersion into PBS in an upside–down position for 10–20 min.

Sections were then incubated for 1 h at room temperature with pre‐conjugated primary antibodies diluted in PBS with 3% BSA and washed 3 times for 5 min in PBS. Slides were again coverslipped, and the first imaging round, including 3 antibodies and DAPI was acquired on the Cell DIVE system at 20x magnification. After imaging, slides were again de‐coverslipped, and fluorophores were bleached by immersion into a solution of 10 mL 0.5 m NaHCO_3,_ 5 mL 30% H_2_O_2,_ and 35 mL double‐distilled H_2_O for 15 min 3 times, and again washed in PBS for 5 min 3 times. The background imaging, antibody incubation, and imaging, and fluorophore bleaching procedures were then repeated until all antibodies had been imaged and integrated into the Cell DIVE system.

### Immunofluorescence Microscopy Analysis and Quantification

Quantification of immunostaining markers was performed on stitched images of the entire tissue sections. A wide panel of markers was chosen to address tissue vasculature (CD31, α‐SMA, VCAM1, ANGPTL4), inflammation (CD163, IL1R, TLR4), proteoglycans (PRG4), and various cell types (NOTCH3, CD90, CD146). Quantitative analyses were performed with the software Fiji (v2.14.0) by thresholding the specific antibody signal (based on signal intensity in negative control images) and quantifying the area. The area with a positive signal was then expressed as a percentage of the total tissue section area. Analysis and quantification were performed while blinded to the samples subject grouping.

Cell DIVE was utilized to stain for a wide selection of proteins on the same tissue sections, allowing both spatial and qualitative exploration of various cell types and tissue structures, including blood vessels. Since this platform did not allow for high throughput of tissue slides, samples from two subjects within each subject group were selected for investigation. As the patellar tendon samples were collected and imaged bilaterally on the same tissue slides, both the right and left tendon samples from the available subjects were analyzed, allowing for more representative data. Unfortunately, a software error resulted in insufficient image quality for one of the ET samples, leaving only one subject in this group. Since both CT subjects displayed bilateral tendinopathy, while the single ET subject displayed unilateral tendinopathy, 4 samples of symptomatic CT, 1 sample of symptomatic ET, 1 sample of presumably healthy contralateral tendon to the ET sample, and 4 samples of healthy CTRL were available. Vasculature‐related proteins, including CD31, VWF, NOTCH3, α‐SMA, and CD146 were used to classify the vessels into distinct phenotypes (Figure S8A, Supporting Information). Using the image analysis software Qupath (v.0.5.1),^[^ ^76^ ^]^ vessel size (area) and density (vessels/tissue area) were examined and compared both across the individual phenotypes and between the disease groups. Based on the observed size difference, the various vessel phenotypes were categorized into either small (200–2000 µm^2^) or large (3000–23000 µm^2^) phenotypes and compared these between tendinopathic and healthy tendons. This size cutoff allowed enough phenotypes in each group to constitute meaningful averages. Cell classification within the perivascular regions was next performed to characterize potential interactions between vascular and non‐vascular cells (Figure S8B, Supporting Information). Since macrophages^[^ ^18^, ^77^ ^]^ and CD90^+^ fibroblast^[^ ^57^, ^78^ ^]^ have been implicated in inflammatory conditions, including tendinopathy, these cell types were focused on. Since the adaptive immune system has been implicated in tendon injury,^[^ ^61^, ^62^ ^]^ It was also investigated if CD3^+^ T cells were present in the tendon tissue, blood vasculature, or lymphatic (PDPN^+^) vasculature.

### Proteomic Analysis

Tissue sections were resuspended in 4% Sodium Dodecyl Sulfate (SDS) in PBS, homogenized by heating for 10 min at 95 °C, and sonicated using a Bioruptor sonicator (settings: 21 °C water with 10 cycles of 30 s on and 30 s off). For de‐crosslinking, samples were heated for 1.5 h at 70 °C. Lysates were cleared by centrifugation for 10 min at 16000 g, and protein concentrations were determined using the Pierce Protein Assay (Thermo Scientific). Subsequently, 4 µg of protein extract was reduced and alkylated for 10 min at 70 °C using 5 mm Tris(2‐carboxyethyl) phosphine hydrochloride (TCEP) and 15 mm chloracetamide (CAA). Protein digestion was performed following the SP3 protocol.^[^ ^79^ ^]^ In brief, 4 µg each of hydrophilic and hydrophobic beads were added to the sample and bound by adding an equal volume of acetonitrile (ACN). After 8 min of incubation, magnetic beads were immobilized and washed twice with 70% ethanol and ACN. Proteins were digested with trypsin (substrate: enzyme ratio 100:1) overnight at room temperature. Samples were acidified to 0.1% formic acid (FA) and cleaned up using house‐made SDB‐RPS tips. For peptide separation, an in‐house‐made fused silica emitter (75 µm diameter, length 15‒30 cm) was packed with 5 µm C18 Poroshell resin (Agilent, USA) and applied to an Ultimate 3000 (Thermo‐Fisher Scientific, Waltham, USA). The column temperature was maintained at 50 °C using an integrated column oven. Peptides were separated using a binary buffer system of buffer A (0.1% formic acid (FA) and buffer B (80% ACN, 0.1% FA on a 30 cm in‐house‐packed analytical column filled with 1.9 µm C18‐AQ Reprosil Pur beads (Dr. Maisch, Germany). A 60‐min segmented gradient of 4–32% buffer B over 43 min, 32–55% buffer B over 7 min, and 55–95% buffer B over 10 min at a flow rate of 300 nL min^−1^ was applied to elute peptides. Peptides were measured with a timsTOF Pro 2 using a CaptiveSpray source (Bruker). Samples were measured in dia‐PASEF mode with daily ion mobility calibration using three ions of Agilent ESI‐Low Tuning Mix following vendor specifications. The DIA‐PASEF window ranged in dimension 1/k0 0.7‐1.45, with 2 ion mobility windows and 12 segments in dimension m/z from 350 to 1250. The mass spectrometry proteomics data have been deposited to the ProteomeXchange Consortium via the PRIDE partner repository with the dataset identifier PXD062414.^[^ ^80^ ^]^ Files were processed with DIA‐NN 1.8.1 using a library‐free search against UniProt Homo Sapiens (2019), complemented with protein sequences from myosin heavy chain variants. Mass ranges were set according to the mass spectrometer settings, with a mass deviation of 15 ppm for files acquired by Bruker instruments. Further calculations were performed within R (version 4.2.2) using the following libraries: diann, tidyverse, data.table, samr, vsn, ggplot2, gprofiler, and missForest. Data was further processed using an in‐house modified R‐script based on the version by V. Demichev (Github page, cit MaxLFQ). Data input was filtered for unique peptides, *q*‐value <0.01, Lib.*Q*.value < 0.01, PG.*Q*.value < 0.01, Global.*Q*.value < 0.01, and Quantity.Quality > 0.7, with Fragment.count ≥ 4. Quality control of iRT peptides was performed in Skyline‐Daily (v22.21.391). Further calculations were performed in R using the packages openxlsx, vsn, limma, corrplot, ggplot2, statmod, and gprofiler. Missing values were imputed using a random forest algorithm for groups with 70% data completeness; for groups with more than 30% missing values, the random forest algorithm was adjusted with a downshift of 0.3 and a width of 1.5. Sex, BMI, and age‐related effects were identified and eliminated using linear models for microarray data (LIMMA). Further analysis was performed in Perseus (v1.6.5.0) and InstantClue (v0.10.10.20211105). ANOVA and Welch's *t*‐test analyses were performed with an FDR of less than 0.05 and 500 randomizations. GO term analysis was performed on significantly changed proteins (FDR < 0.05) using the R package gprofiler. Also, the online database MatrisomeDB 2.0^[^ ^29^ ^]^ was searched to identify proteins related to the extracellular matrix.

### Statistical Analyses

Proteomic data analysis is described in the proteomics section. The remaining analyses were performed using GraphPad Prism (v. 10.4.1). Data from all available participants were included in the various statistical comparisons, however, participant number varies between different methodological approaches due to corrupted scan files or insufficient biopsy material during experiments. Data normality was tested using the Shapiro–Wilk and Anderson‐Darling tests. Data with normal distribution is presented as mean ± SD and analyzed using unpaired t‐tests, one‐way ANOVA, or two‐way ANOVA with repeated measures for leg. Holm‐Šídák and Šídák multiple comparisons test was used for post‐hoc tests, respectively. Non‐normally distributed data is presented as median and interquartile range and analyzed using Mann–Whitney or Kruskal‐Wallis test with Dunn's multiple comparisons test (using a Bonferroni correction). Pearson and non‐parametric Spearman tests were used to investigate correlations for normally and non‐normally distributed data, respectively. The specific tests are stated in the figure legends. For the main group comparisons, one healthy tendon was chosen using a randomization algorithm for the CTRL group and used for all analyses except the Doppler flow comparison, in which the healthy tendon with the most Doppler flow was used. For ET and CT, the symptomatic tendon was included for unilateral cases, and the most symptomatic tendon was included for bilateral cases. Additional group comparisons were examined, including both right and left healthy tendons in CTRL and both tendons for bilateral tendinopathies in ET and CT, providing a higher sample size. For group comparisons of proteomic data, both right and left healthy tendons were included for each participant in CTRL, while only the most symptomatic tendon was included in ET and CT. For Cell DIVE analyses, the low number of samples precluded statistical testing. P values between 0.05–0.1 are presented in figures with numerical values, while *p* < 0.05 was considered statistically significant and presented in figures with asterisks.

## Conflict of Interest

The authors declare no conflict of interest.

## Author contributions

MFRM, NMM, HRS, SPM and MK contributed to the conception of the study. MFRM, NMM, ML, RBS, and CCY contributed to patient recruitment and data collection. MFRM, RBS, SGD, MK, LS, and JA contributed to lab work and data analysis. MFRM, RBS, SGD, MK, LS, JA, CCY, SPM and MK contributed to data interpretation and discussion. All authors contributed to manuscript writing and approved the final draft.

## Supporting information



Supporting Information

## Data Availability

The data supporting the findings in this study are presented in the supporting information and deposited to the ProteomeXchange Consortium via the PRIDE partner repository with the dataset identifier PXD062414. Further data are available from the authors upon reasonable request.
