# Supplementary material for: Increased Blood Flow and Tendon Swelling Precedes Vascular Expansion and Tissue Matrix Changes In Early Human Tendinopathy: A Potential Window for Superior Treatment Response
Source: Adv Sci (Weinh). 2025 Dec 12;13(12):e14023. doi: 10.1002/advs.202514023 (PMC12948216; doi:10.1002/advs.202514023)
Supplement: Supplementary file 1 — Supporting Information [file ADVS-13-e14023-s001.docx]

**Title:** Increased blood flow and tendon swelling precedes vascular expansion and tissue matrix changes in early human tendinopathy: A potential window for superior treatment response

**Authors:** Max FR. Merkel*, Nikolaj M. Malmgaard-Clausen, Marius Lendal, Hartwig R. Siebner, René B. Svensson, Stephanie G. Dakin, Marcus Krüger, Luisa Schmidt, Jakob Agergaard, Ching-Yan Chloé Yeung, S. Peter Magnusson, Michael Kjaer

*Corresponding author. Email: [max.flemming.ravn.merkel@regionh.dk](mailto:max.flemming.ravn.merkel@regionh.dk)

**List of Supplementary Materials**:

**Figure S1.** MRI tendon dimensions and pixel intensity.

**Figure S2.** Group comparisons of MRI tendon dimensions including bilateral samples.

**Figure S3.** Tendon blood flow and symptoms correlations.

**Figure S4.** Immunofluorescence of healthy and tendinopathic tendons.

**Figure S5.** Immunofluorescent staining and quantification including bilateral samples.

**Figure S6.** Tendon vasculature in asymptomatic tendinopathy.

**Figure S7.** Cell DIVE multiplex images.

**Figure S8.** Cell DIVE image analysis.

**Figure S9.** Cell DIVE images of blood vessel organization

**Table S1.** Cell DIVE image analysis of blood vessel phenotypes showing individual participant and group data.

**Table S2.** List of antibodies used for multiplex and Cell DIVE histology.

**Table S3.** Summary table of tendon samples available for the various methods utilized.

**Figure S1. MRI tendon dimensions and pixel intensity.** (**A)** Correlation between proximal patellar tendon CSA measured with 3T and 7T MRI and individuals body mass in the CTRL group including both right and left tendons. Group comparisons of patellar tendon **(B)** volume and **(C)** proximal CSA between CTRL and the asymptomatic tendons of participants with unilateral tendinopathy in ET and CT. **(D)** Group comparison of patellar tendon regional MRI pixel intensity in healthy controls (CTRL), early (ET) and chronic (CT) tendinopathy measured using 3T and 7T MRI. Group comparisons include only one tendon per participant. Data shown as individual datapoints with mean ﻿± SD or median and IQR for data with normal and non-normal distribution, respectively. Correlations of pain scores and symptom duration with **(E)** proximal patellar tendon CSA relative to body mass and **(G)** proximal pixel intensity including ET and CT participants. For SLDS-NRS and symptom duration n = 32-34 since both tendons were included for participants with bilateral tendinopathy, while n = 26-27 for activity pain, morning pain and VISA-P since these were only reported for the most symptomatic tendon. Correlations tested using Pearson correlation coefficient in (A). One-way ANOVA with Holm-﻿Šídák correction performed in (B,C, D (7T)). Kruskal-Wallis with Dunn’s multiple comparisons test performed in (D (3T)). Correlations tested using Spearman correlation coefficient in (E, F).

*P<0.05, **P<0.01, ***P<0.001. NRS, numeric rating scale; SLDS, single-leg decline squat; CSA, cross sectional area; Asymp, asymptomatic; AU, arbitrary unit.

**Figure S2. Group comparisons of MRI tendon dimensions including bilateral samples.** Group comparisons between healthy controls (CTRL), early (ET) and chronic (CT) tendinopathy of (**A)** Patellar tendon volumes, **(B)** proximal tendon CSA relative to body mass and **(C)** regional tendon CSA measured using 3T and 7T MRI. For all comparisons both the right and left patellar tendon was included for the CTRL group, and in ET and CT both tendons were included for participants with bilateral tendinopathy. Data shown as individual datapoints with mean ﻿± SD. P values represent one-way ANOVA with Holm-﻿Šídák correction. *P<0.05, **P<0.01,***P<0.001, ****P<0.0001. CSA, cross sectional area; Bi, bilateral.

**Figure S3. Tendon blood flow and symptoms correlations.** **(A)** Spearman correlations of tendon Doppler flow with VISA-P and symptom duration. For symptom duration n = 39 since both tendons were included for participants with bilateral tendinopathy, while for VISA-P n = 30 since these were only reported for the most symptomatic tendon. **(B)** Within participant comparisons of symptomatic and non-symptomatic tendon and peritendon Doppler flow in participants with unilateral early (ET) and chronic (CT) tendinopathy. Data shown as paired individual datapoints for right and left tendons within the same participant. **(C)** Group comparisons of tendon and peritendon Doppler flow including both the right and left patellar tendon in CTRL and for participants with bilateral tendinopathy in ET and CT. Data shown as individual datapoints with median and IQR. Two-way repeated measures ANOVA and ﻿Šídák multiple comparisons test performed in (B). Kruskal-Wallis with Dunn’s multiple comparisons test performed in (C). *P<0.05, **P<0.01,***P<0.001, ****P<0.0001. ME, main effect of two-way ANOVA; Group x Leg = interaction in two-way ANOVA.


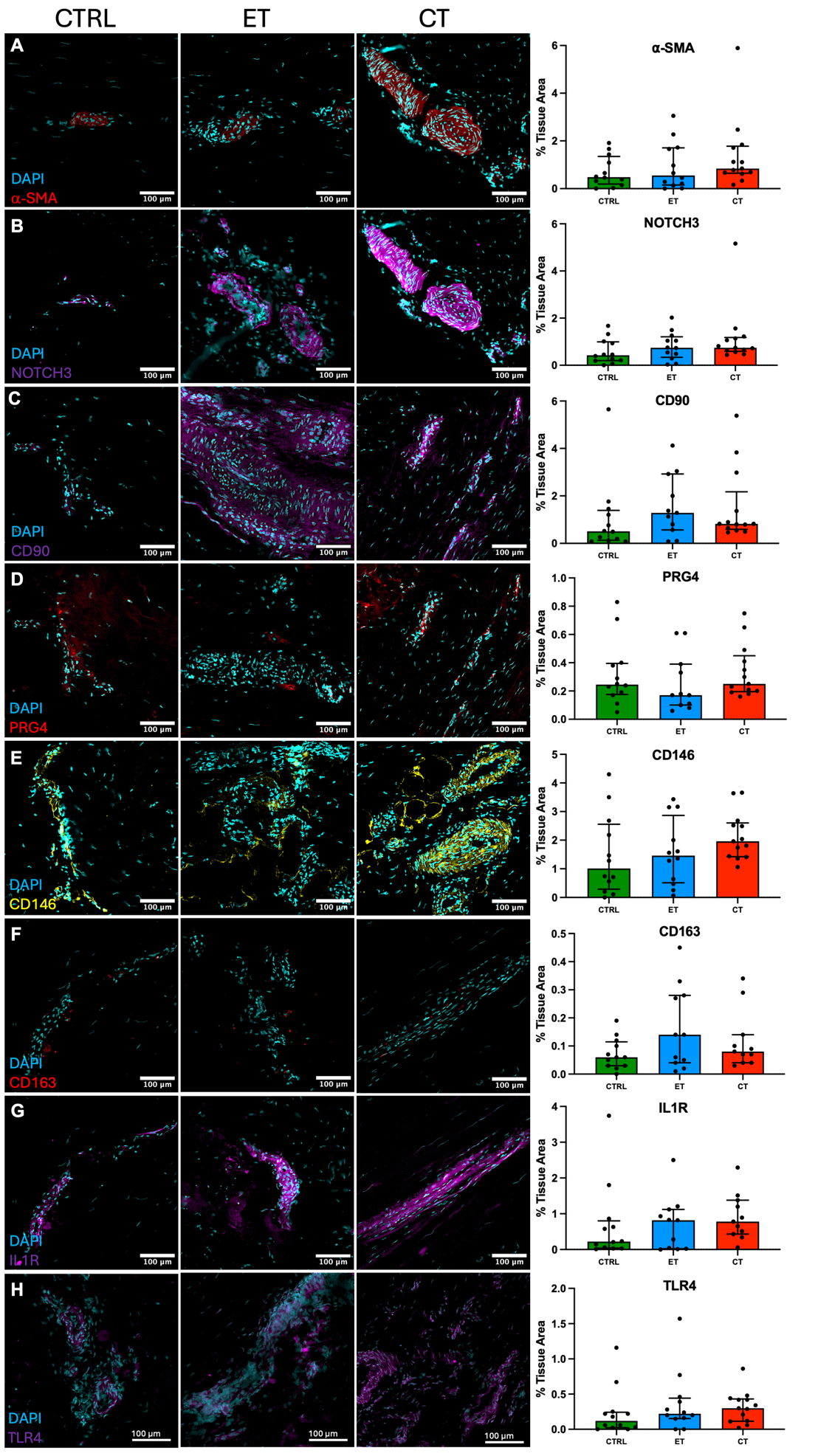


**Figure S4. Immunofluorescence of healthy and tendinopathic tendons.** Immunofluorescent staining and quantification of **(A)** α-SMA, **(B)** NOTCH3, **(C)** CD90, **(D)** PRG4, **(E)** CD146, **(F)** CD163, **(G)** IL1R and **(H)** TLR4 in healthy (CTRL), early (ET) and chronically (CT) tendinopathic tendons. Group comparisons include only one tendon per participant. Data shown as individual datapoints with median and IQR. Group differences tested using Kruskal-Wallis test.

**Figure S5. Immunofluorescent staining and quantification including bilateral samples.** Group comparisons between healthy (CTRL), early (ET) and chronically (CT) tendinopathic tendons of immunofluorescence markers. For all comparisons both the right and left patellar tendon sample was included for the CTRL group, and in ET and CT both samples were included for participants with bilateral tendinopathy. Data shown as individual datapoints with median and IQR. Group differences tested using Kruskal-Wallis with Dunn’s multiple comparisons test. *P<0.05,***P<0.001. Bi, bilateral.

**Figure S6. Tendon vasculature in asymptomatic tendinopathy.** **(A)** Spearman correlation including all symptomatic ET and CT tendons of Doppler flow and CD31 immunofluorescence quantification demonstrating that microscopically observed vasculature in tendon biopsies correlated with tendon Doppler blood flow. **(B)** Group comparisons between healthy (CTRL) and asymptomatic tendons of participants with unilateral tendinopathy in ET and CT. ET and CT were pooled due to the low number of available samples. Group comparisons include only one tendon per participant. Data shown as individual datapoints with mean ﻿± SD or median and IQR for data with normal and non-normal distribution, respectively. Mann-Whitney test performed in (B (nuclei, VCAM1, VEGF), and unpaired t-test performed in (B (CD31)). *P<0.05. Asymp, asymptomatic.

**Figure S7. Cell DIVE multiplex images.** Overview images of blood vessel phenotypes and various cell types in healthy (CTRL), early (ET) and chronic (CT) tendinopathy.

**Figure S8. Cell DIVE image analysis.** **(A)** Images illustrating vessel phenotype classification including segmentation of vessels based on all vascular markers combined followed by pixel classification of vessel segments for each individual marker. **(B)** Images illustrating vascular and perivascular cell classification including DAPI-based cell detection and subsequent cell classification using various vascular markers. Analysis was performed using software QuPath (v.0.5.1).


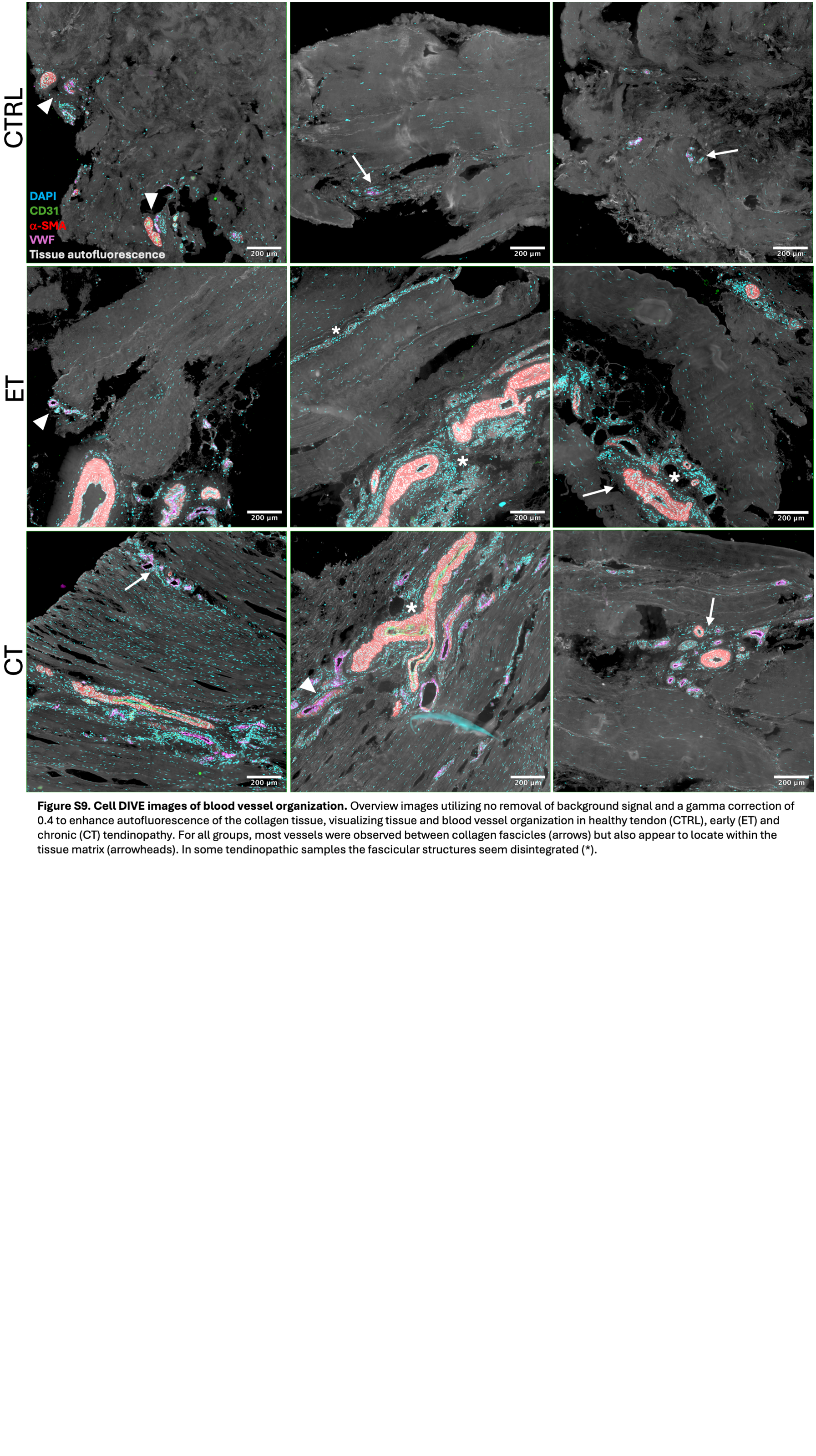


**Figure S9. Cell DIVE images of blood vessel organization.** Overview images utilizing no removal of background signal and a gamma correction of 0.4 to enhance autofluorescence of the collagen tissue, visualizing tissue and blood vessel organization in healthy tendon (CTRL), early (ET) and chronic (CT) tendinopathy. For all groups, most vessels were observed between collagen fascicles (arrows) but also appear to locate within the tissue matrix (arrowheads). In some tendinopathic samples the fascicular structures seem disintegrated (*).

**Table S1.** Cell DIVE image analysis of blood vessel phenotypes showing individual participant and group data. Blood vessel phenotypes are categorized into either small or large sized phenotypes, and a selection of the most abundantly observed phenotypes are shown individually**.** N/A indicates that vessel phenotype was not observed in the sample. N/A, not applicable; CT, chronic tendinopathy; ET, early tendinopathy; Asymp, asymptomatic; CTRL, healthy control; avg, average; L, left; R, right.

**Table S2.** List of antibodies used for multiplex and Cell DIVE histology.

**Table S3.** Summary table of number of tendon samples available for the various methods utilized. For main comparisons only the most symptomatic tendon vas included for each participant. Further comparisons were tested including both healthy tendons for CTRL and both symptomatic tendons for participants with bilateral tendinopathy. Proteomic data was analyzed including bilateral samples for CTRL and unilateral samples for ET and CT. CTRL, healthy controls; ET, early tendinopathy; CT, chronic tendinopathy; N/A, not applicable.

| Method | Available samples | | | Available including bilateral samples | | | Reason for samples missing |
| --- | --- | --- | --- | --- | --- | --- | --- |
|  | CTRL | ET | CT | CTRL | ET | CT |  |
| Ultrasound Doppler | 15 | 14 | 16 | 30 | 16 | 23 | All participants included |
| 3T MRI | 13 | 14 | 14 | 26 | 16 | 19 | MRI data missing/lost |
| 7T MRI | 14 | 13 | 16 | 28 | 15 | 23 | MRI data missing/lost |
| Biopsy staining | 12 | 12 | 13 | 23 | 13 | 18 | Insufficient biopsy material |
| Biopsy Proteomics | N/A | N/A | N/A | 20 | 11 | 12 | Insufficient biopsy material |
